# Amygdala-predominant α-synuclein pathology is associated with exacerbated hippocampal neuron loss in Alzheimer’s disease

**DOI:** 10.1093/braincomms/fcae442

**Published:** 2024-12-05

**Authors:** Klara Gawor, Sandra O Tomé, Rik Vandenberghe, Philip Van Damme, Mathieu Vandenbulcke, Markus Otto, Christine A F von Arnim, Estifanos Ghebremedhin, Alicja Ronisz, Simona Ospitalieri, Matthew Blaschko, Dietmar Rudolf Thal

**Affiliations:** Laboratory for Neuropathology, Department of Imaging and Pathology, KU Leuven, Leuven 3000, Belgium; Laboratory for Neuropathology, Department of Imaging and Pathology, KU Leuven, Leuven 3000, Belgium; Laboratory for Cognitive Neurology, Department of Neurosciences, KU Leuven, Leuven 3000, Belgium; Department of Neurology, University Hospitals Leuven, Leuven 3000, Belgium; Department of Neurology, University Hospitals Leuven, Leuven 3000, Belgium; Laboratory for Neurobiology, Department of Neuroscience, KU Leuven, Leuven 3000, Belgium; Laboratory for Translational Neuropsychiatry, Department of Neuroscience, KU Leuven, Leuven 3000, Belgium; Department of Neurology, Ulm University, Ulm 89081, Germany; Department of Neurology, Martin Luther University Halle-Wittenberg, Halle 06120, Germany; Department of Neurology, Ulm University, Ulm 89081, Germany; Department of Geriatrics, University Medical Center Göttingen, Göttingen 37073, Germany; Institute for Clinical Neuroanatomy, Johann Wolfgang Goethe University, Frankfurt am Main 60596, Germany; Laboratory for Neuropathology, Department of Imaging and Pathology, KU Leuven, Leuven 3000, Belgium; Laboratory for Neuropathology, Department of Imaging and Pathology, KU Leuven, Leuven 3000, Belgium; Processing Speech and Images, Department of Electrical Engineering, KU Leuven, Leuven 3000, Belgium; Laboratory for Neuropathology, Department of Imaging and Pathology, KU Leuven, Leuven 3000, Belgium; Department of Pathology, University Hospitals Leuven, Leuven 3000, Belgium

**Keywords:** dementia, neuropathology, path analysis, Lewy body disease, immunohistochemistry

## Abstract

Misfolded α-synuclein protein accumulates in 43–63% of individuals with symptomatic Alzheimer’s disease. Two main patterns of comorbid α-synuclein pathology have been identified: caudo-rostral and amygdala-predominant. α-Synuclein aggregates have been shown to interact with the transactive response DNA-binding protein 43 (TDP-43) and abnormally phosphorylated tau protein. All these proteins accumulate in the amygdala, which is anatomically connected with the hippocampus. However, the specific role of amygdala-predominant α-synuclein pathology in the progression of Alzheimer’s disease and hippocampal degeneration remains unclear. In this cross-sectional study, we analysed 291 autopsy brains from both demented and non-demented elderly individuals neuropathologically. Neuronal density in the CA1 region of the hippocampus was assessed for all cases. We semiquantitatively evaluated α-synuclein pathology severity across seven brain regions and calculated a ratio of limbic to brainstem α-synuclein pathology severity, which was used to stratify the cases into two distinct spreading patterns. In the 99 symptomatic Alzheimer’s disease cases, we assessed severity of limbic-predominant age-related TDP-43 neuropathological changes and CA1 phosphorylated tau density. We performed triple fluorescence staining of medial temporal lobe samples with antibodies against phosphorylated TDP-43, α-synuclein and phosphorylated tau. Finally, we employed path analysis to determine the association network of various parameters of limbic pathology in Alzheimer’s disease cases and CA1 neuronal density. We identified an association between the amygdala-predominant αSyn pathology pattern and decreased neuronal density in the CA1 region. We found that Alzheimer’s disease cases with an amygdala-predominant α-synuclein pattern exhibited the highest TDP-43 severity and prevalence of TDP-43 inclusions in the dentate gyrus among all groups, while those with the caudo-rostral pattern had the lowest severity of Alzheimer’s disease neuropathological changes. We observed colocalization of TDP-43, aggregated α-synuclein and hyperphosphorylated tau in cytoplasmic inclusions within hippocampal and amygdala neurons of Alzheimer’s disease cases. Path analysis modelling suggests that the relationship between amygdala-predominant α-synuclein pathology and CA1 neuron loss is partially mediated by hippocampal tau and TDP-43 aggregates. Our findings suggest that Alzheimer’s disease cases with amygdala-predominant α-synuclein pathology may constitute a distinct group with more severe hippocampal damage, a higher TDP-43 burden and potential interactions among α-synuclein, TDP-43 and hyperphosphorylated tau.

## Teaser

Gawor *et al*. studied Alzheimer’s disease cases with Lewy body pathology exhibiting two spreading patterns. The amygdala-predominant pattern was linked to more severe hippocampal damage than the caudo-rostral pattern. These cases also had more extensive transactive response DNA-binding protein 43 pathology, with frequent tau, transactive response DNA-binding protein 43 and α-synuclein aggregates collocalizing in the limbic regions.

## Graphical Abstract

**Graphical Abstract:**
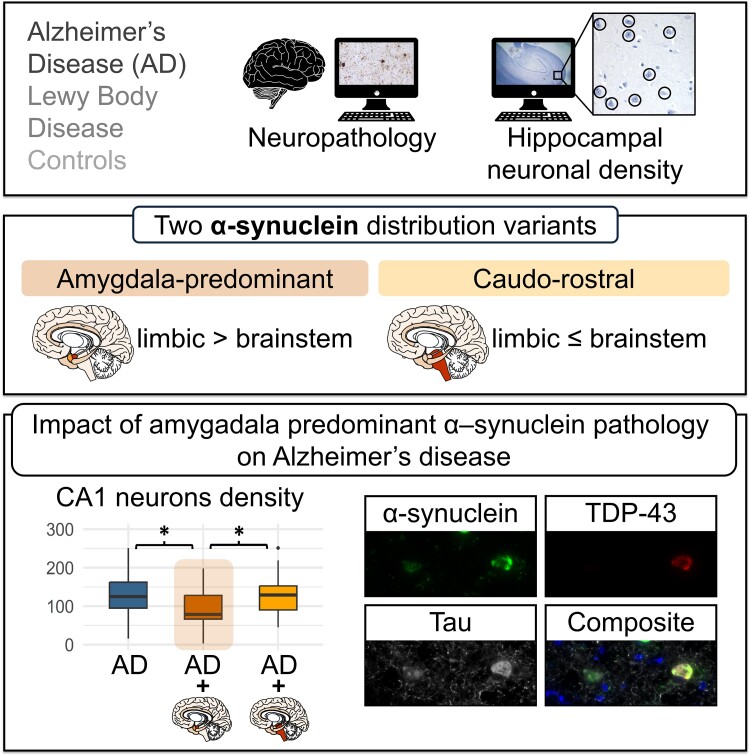

## Introduction

α-Synuclein (αSyn) is a protein abundantly present in the human brain, primarily localized within pre-synaptic terminals under normal physiological conditions.^1^ The accumulation of misfolded αSyn in the grey matter is a significant contributor to age-related brain degeneration, clinically manifested as Lewy body disorders (LBD), which encompass Parkinson’s disease, Parkinson’s disease dementia and dementia with Lewy bodies.^2^ In LBD, αSyn aggregates can take the form of Lewy bodies, Lewy neurites and astrocytic inclusions, depending on their specific locations.^3^ Remarkably, similar morphological αSyn pathology has been detected in 43–63% of individuals diagnosed with Alzheimer’s disease.^4-7^

In advanced cases with αSyn pathology, the aggregates are observed in multiple brain regions, including the brainstem, limbic system and neocortex.^8-11^ Although αSyn pathology in many affected individuals follows a typical pattern of distribution described by Braak *et al*.,^9^ considerable heterogeneity exists in the severity across brain regions. Various attempts to identify underlying spreading patterns have been made.^12-15^ Despite some variations, a consistent trend has emerged, indicating that some αSyn-positive cases exhibit early involvement of the amygdala (amygdala-predominant), while in others, the first brain lesions appear in the lower brainstem areas (caudo-rostral) with early or late involvement of the olfactory bulb.^12-15^

Amygdala-predominant αSyn pathology is often associated with comorbid Alzheimer’s disease neuropathological changes (ADNC),^7,13,14,16^ which includes amyloid β (Aβ) plaques, neuritic plaques and neurofibrillary tangles (NFTs) consisting of abnormal phosphorylated tau protein (pTau). Within the spectrum of Alzheimer’s disease, various subtypes have been identified based on the severity of NFT pathology including limbic-predominant, hippocampal sparing and typical Alzheimer’s disease.^7^ Among these, the limbic-predominant subtype is characterized by more severe limbic system pathology compared with that in the neocortex^17^ and has been recently identified to have the highest occurrence of αSyn pathology in the amygdala.^3^

Hippocampal degeneration is one of the hallmarks of Alzheimer’s disease, affecting about 80% of Alzheimer’s disease patients, though significant variability in the extent of degeneration is observed among individuals.^18^ The limbic-predominant subtype of Alzheimer’s disease was shown to be characterized by the most severe volume loss of that region.^19^ Both the APOE ε4 variant^20^ and pTau pathology^21^ have been linked with increased hippocampal atrophy, and numerous studies also highlight the significant impact of transactive response DNA-binding protein 43 (TDP-43).^22-24^ Furthermore, cell loss in the CA1 region has been linked to arteriolosclerosis,^25^ cerebral amyloid angiopathy (CAA),^26,27^ epilepsy,^28^ as well as metabolic imbalances such as ischaemia and hypoglycaemia.^29^ This demonstrates that hippocampal degeneration is a multifaceted phenomenon, potentially influenced by different sets of factors in different individuals.

Primate studies using tracer injections have demonstrated anatomical connectivity between the amygdaloid complex and hippocampal regions, particularly CA1 and subiculum.^30-32^ The neuron-to-neuron spread of pTau and αSyn between anatomically connected areas has long been speculated due to the hierarchical progression of these pathologies.^9,33^ Supporting evidence includes animal studies showing the spread of αSyn and tau fibrils to connected regions,^34-37^ tau *in vivo* imaging models^38,39^ and the possibility of synaptic transmission of tau seeds,^40-42^ although non-synaptic mechanisms involving glia have also been proposed.^43^ Thus, it can be hypothesized that the amygdala plays a crucial role in spreading pathological aggregates to the hippocampus.

In patients with LBD, αSyn pathology primarily affects the CA2 region.^44^ Despite the severity of αSyn pathology in CA1 correlating with memory impairment in LBD,^45^ studies investigating the CA1 and subiculum regions have found no clear effects of αSyn pathology on the degeneration of these regions.^46,47^ In Alzheimer’s disease, αSyn pathology coexists with other protein aggregates. Numerous studies have demonstrated the potential of αSyn to interact with pTau,^48,49^ Aβ^50,51^ and TDP-43.^52-54^ Furthermore, the amygdala-predominant variant of αSyn is more commonly observed in Alzheimer’s disease,^13^ raising important questions about its potential involvement in CA1 region degeneration.

In this study, we investigated whether αSyn pathology influences the degeneration of the CA1 region of the hippocampus similarly to other Alzheimer’s disease-related pathologies. We categorized αSyn pathology into amygdala-predominant and caudo-rostral variants, hypothesizing that the hippocampal degeneration patterns might vary among these αSyn pathology subtypes in Alzheimer’s disease cases. To test this hypothesis, we evaluated the differences in neuropathological profiles of Alzheimer’s disease cases with these two patterns of αSyn pathology distribution and carried out multivariate statistical analyses to identify the association between αSyn variant, pTau, phosphorylated TDP-43 (pTDP-43) pathology and hippocampal neuron loss.

## Materials and methods

### Human samples

We examined 291 autopsy brains obtained from university or municipal hospitals in Leuven (Belgium, Research Ethics Committee UZ/KU Leuven identifiers: S52791, S55312, S59292 and S64363), Bonn, Offenbach am Main and Ulm (Germany, Ethics Committee UZ/KU Leuven identifier: S59295 and Ulm identifier: 58/08) and from GE-Healthcare (ClinicalTrials.gov identifiers NCT01165554 and NCT02090855). All of the experiments have been performed after ethical clearance by the UZ/KU Leuven Ethics Committee (S65147). The brain samples were collected following local legislation and federal laws governing the use of human tissue for research in these three countries. Clinical files have been analysed for the presence of parkinsonism symptoms, diagnosis of epilepsy and dementia status. The degree of dementia at the time of death was determined retrospectively using an estimate of the Clinical Dementia Rating global score^55^ and/or Mini-Mental Score Examination.^56^ The Mini-Mental Score Examination has been converted to a Clinical Dementia Rating score using published cut-off values.^57^ A description of the neuropathological and clinical characteristics of the study group is given in Supplementary Table 1.

Exclusion criteria listed in Supplementary Table 2 were applied to ensure the homogeneity of the study population. While cases with a clinical diagnosis of seizure disorder were not excluded, this information was utilized as a confounding factor in our primary analysis. In cases featuring TDP-43 proteinopathies, only cases with clear features of either amyotrophic lateral sclerosis or frontotemporal lobar degeneration with TDP-43-immunoreactive pathology subtypes (diagnosed according to the published criteria)^58^ were excluded, owing to the common co-occurrence of pTDP-43 pathology in Alzheimer’s disease.^23,59^

### Immunohistochemistry

Formalin-fixed tissue samples from either the left (*n* = 245) or the right brain hemisphere (*n* = 46), including the anterior medial temporal lobe (MTL), posterior MTL with the hippocampus, middle frontal gyrus, occipital cortex, midbrain, pons and medulla oblongata, were used in this study. The tissue was embedded in paraffin and sectioned into 5-μm-thick sections using a microtome (Thermo Fisher Scientific). Immunohistochemistry was employed to stain these sections. Detailed information about the antibodies used and the regions stained is given in Supplementary Table 3. A robotic autostainer (Leica Microsystems) was used to perform tissue deparaffinization. Antigen retrieval was carried out in a PT Link Module (Dako) using EnVision Flex Target Retrieval Solution Low pH (a citrate-based buffered solution with a pH of 6.1, Dako). An additional step involving a 5-min incubation with formic acid was included for αSyn and Aβ staining.

For the staining with 3,3′-diaminobenzidine, we applied EnVision FLEX Peroxidase-Blocking Reagent (Dako) to the tissue for 5 min to block endogenous peroxidase activity. Tissue sections were incubated overnight in a humid chamber with the primary antibody. The next day, anti-mouse horseradish peroxidase-linked secondary antibodies were used for staining against pTau and αSyn, while the anti-rabbit VectaStain ABC-HRP kit (Vector Laboratories) was employed for pTDP-43 staining to enhance the signal. Finally, 3,3′-diaminobenzidine solution (liquid 3,3′-diaminobenzidine + substrate–chromogen system, Dako) was used to visualize the binding between the primary antibody and secondary antibody. All slides were counterstained with haematoxylin using an autostainer, and mounting was performed with an automated coverslipper (Leica Microsystems).

Triple labelling immunofluorescence staining was conducted on posterior MTL slides with hippocampus and anterior MTL slides with amygdala from five cases with a clinical diagnosis of dementia, severe ADNC, pTDP-43 positivity and amygdala-predominant αSyn pathology. The tissue was incubated with formic acid for 5 min, followed by overnight incubation with a cocktail of primary antibodies: anti-pTDP-43 (409/410, Cosmo Bio, dilution 1:1000, rabbit) and anti-aggregated αSyn (5G4, Merck Millipore, dilution 1:500, mouse). Subsequently, the sections were treated with fluorescent secondary antibodies: donkey anti-rabbit conjugated with Cy3 and donkey anti-mouse conjugated with Cy2 (Jackson ImmunoResearch). Following this, we applied a biotinylated anti-pTau^S202/T205^ antibody (AT8, Thermo Fisher, dilution 1:500). The binding of the biotinylated antibody was visualized using fluorochrome-conjugated streptavidin (Cy5, Jackson ImmunoResearch, dilution 1:50). We applied TrueBlack Lipofuscin Autofluorescence Quencher (Biotium) to avoid interference with autofluorescence signal. The slides were mounted with ProLong Gold Antifade Mountant containing DAPI (Invitrogen) for nuclear counterstaining.

### Pathology assessment and quantification

A ZEISS Axio Imager 2 microscope equipped with an Axiocam 506 camera and a DM2000 LED Leica microscope equipped with a Leica DFC7000 T digital camera were used for the examination and digital photography of the tissue. The overview of all parameters used in this study and their operationalization is given in Supplementary Table 4.

Pathological assessment was conducted for all cases following established protocols. This included evaluating Braak stages of NFT progression,^33^ phases of Aβ deposition in MTL^60^ and CERAD scores for neuritic plaques.^61^ The Braak NFT stages, Aβ MTL phases and CERAD scores were translated into A, B and C scores, respectively.^62^ An ‘ABC score’ reflecting the severity of ADNC was calculated according to the National Institute on the Alzheimer’s Association guidelines.^62^ We assessed the CAA type (CAA type 1 with capillary involvement versus CAA type 2 lacking capillary involvement) and Vonsattel grade for the severity of CAA.^63,64^ Additionally, we assessed the presence of pTDP-43 lesions in the posterior MTL and dentate gyrus, the presence of argyrophilic grain disease (AGD)^65^ and aging-related tau astrogliopathy (ARTAG)^66^ in the MTL using pTau (AT8) staining. The presence of haemorrhagic and ischaemic infarcts was determined macroscopically by evaluating brain slices and microscopically by analysing haematoxylin–eosin sections as part of the diagnostic process. Cerebral small vessel disease (SVD) was characterized by intimal deterioration, smooth muscle degeneration and lipohyalinotic or hyaline thickening of arterioles and small arteries with or without consequent narrowing of the vascular lumen.^67^ SVD in the white matter of the temporal cortex was assessed using haematoxylin–eosin-stained sections from the posterior and anterior MTL. Severity scores for temporal lobe arteriolosclerosis were assigned based on the percentage of affected vessels as follows: 0: no affected vessels; 1: mild: < 30% affected vessels; 2: moderate: 30–<60% affected vessels; and 3: severe: ≥ 60% affected vessels.

Cases that exhibited αSyn immunoreactivity in the medulla oblongata or anterior MTL slide underwent further screening for αSyn pathology in the pons, midbrain, posterior MTL and frontal cortex to determine the Braak LBD Stage.^9^ Additionally, we evaluated αSyn lesion severity using semi-quantitative criteria as previously outlined^9^ and the third report from the Dementia with Lewy Bodies Consortium.^11^ The details of the analysed regions are presented in Supplementary Table 5. Locus coeruleus in the pons was not assessed for αSyn pathology severity as the tissue was not always available. Our assessment covered all types of αSyn lesions, including Lewy neurites, Lewy bodies and glial inclusions within grey matter. The representative levels and used criteria for each severity category in each brain region are shown in Supplementary Fig. 1. We calculated the *global burden score for αSyn (αSynGBS)* by summing the severity scores across the five brain regions (medulla oblongata, midbrain, amygdala, temporal cortex and frontal cortex). Additionally, we computed the ratio of αSyn pathology severity scores for cases having both limbic and brainstem αSyn pathology as follows: *αSyn limbic/brainstem ratio* = (*amygdala + posterior temporal cortex*)*/*(*medulla oblongata + midbrain*). For cases with αSyn pathology lacking brainstem involvement, the ratio was set to infinity. We used this ratio to stratify cases into two αSyn pathology spreading patterns, considering cases with a ratio higher than one as ‘amygdala-predominant’ and the rest as ‘caudo-rostral’.

Quantification of neurons in the CA1 region was performed on haematoxylin-stained sections of the posterior hippocampus. The border of CA1 and CA2 is defined by an increase in thickness and a decrease in density of the neuronal band. The border between CA1 and the subiculum has been defined by a straight line drawn between the collateral sulcus and the medial part of the fimbria of the hippocampus. Three non-overlapping images of the CA1 region were taken using a magnification of 200 × and an image size of 500 × 500 µm, ensuring that only tissue from the CA1 subfield was included. Whenever possible, photographs were taken from intact tissue sections with uniformly distributed neurons and no large blood vessels. Neurons were selected based on specific criteria: the presence of a spherical nucleus and a visible, often pyramid-shaped, cytoplasm (Supplementary Fig. 2A). Neurons on the edge of a given image that were not fully visible were excluded from counting. Neuron counting was performed manually by a single rater (K.G.) using the QuPath^68^ annotation tool, and the counts were converted to density per mm². To confirm the reliability of this method, 48 randomly chosen cases were also quantified by an independent rater (S.O.T.). We achieved interrater agreement, with a Pearson’s correlation coefficient of 0.84 and a single-score intraclass correlation coefficient of 0.82.

We defined a group of symptomatic Alzheimer’s disease cases as having a clinical diagnosis of dementia with moderate or severe ADNC neuropathological changes (‘ABC’ score 2 or 3). For this group, we additionally assessed the severity of pTau pathology in the CA1 region by taking three photographs of this region from anti-pTau^S202/T205^-stained slides and quantifying the proportion of pTau-positive neurons (neurons with NFTs or pre-tangles) to the total number of neurons (Supplementary Fig. 2B). The selection criteria for neurons were the same as those used in the neuronal density quantification. Additionally, we screened for the presence of pTDP-43 lesions in the amygdala, posterior hippocampus and frontal cortex, and for cases with all three regions available, we classified the limbic-predominant age-related TDP-43 neuropathological changes (LATE-NC) stages using the guidelines published by Nelson *et al*.^69^

### Data analysis

Data analysis was conducted using R Studio software (version 2023.06.0 with R-4.3.1). An a priori power analysis was conducted using G*Power version 3.1.9.7.^70^ For multiple linear regression analyses, we calculated the a priori sample size for our largest model (eight predictors) to achieve 95% power with α = 0.05, detecting a medium effect for R2 deviating from 0. The analysis indicated a minimum sample size of 160, which is less than the 284 cases we utilized. To determine the minimum sample size needed to test differences among three groups of Alzheimer’s disease cases using ANOVA, we found that a sample size of 100 would be required to achieve 95% power for detecting a large effect (*f* = 0.4) at a significance level of α = 0.05. This is nearly identical to the sample size used in our study (*n* = 99).

To investigate relationships with age at death as a covariate, we performed a semi-partial Spearman correlation analysis using the ppcor library.^71^ Specifically, we analysed the following relationships: (i) αSyn global burden score and ADNC, (ii) αSyn limbic/brainstem ratio and ADNC and (iii) αSyn severity in brain regions and CA1 neuronal density. For the correlation analysis between the αSyn limbic/brainstem ratio and ADNC, infinite ratio values were replaced with a large numerical value (1 billion). In cases with a single missing value for αSyn pathology severity in all assessed brain regions, we assumed that αSyn severity forms a sequence, based on the assumption of spreading. Therefore, missing values for the midbrain, amygdala, posterior hippocampus and temporal cortex were imputed using the na.approx function from the zoo library.^72^

We applied the Kruskal–Wallis test followed by the Dunn–Bonferroni *post hoc* test to examine CA1 neuronal density across different cases of αSyn pathology. Subsequently, we conducted a series of multiple linear regression analyses to investigate the relationship between various neuropathological factors and CA1 neuronal density. To determine significant relationships between the three Alzheimer’s disease groups, we employed pairwise Fisher’s exact tests. For differences in continuous variables, we assessed the normality assumption in each group using the Shapiro–Wilk test. When this test was not satisfied, as well as for ordinal variables, we applied the Kruskal–Wallis test with a Dunn–Bonferroni *post hoc* test. For normally distributed variables, we used ANOVA with the Dunn–Bonferroni *post hoc* test. To mitigate the false discovery rate, *P*-values from correlation analyses and all *post hoc* tests were adjusted using the Benjamini–Hochberg method.

Finally, using only data from Alzheimer’s disease cases, we modelled the association between observable neuropathological parameters through path analysis within the structural equation modelling framework (Supplementary Fig. 3, lavaan package version 0.6.16).^73^ Path models enable performing mediation analysis and examining both direct and indirect relationships between multiple variables,^74,75^ a method successfully applied in the study of multifactorial diseases such as diabetes.^76^ We hypothesized that amygdala-predominant αSyn pathology exerts a downstream effect on other variables, based on the literature showing the aggravating properties of αSyn pathology on pTDP-43 and pTau pathology,^53,77-79^ thereby treating it as the sole exogenous variable. The endogenous variables in our model included the percentage of neurons affected by pTau in the CA1 region, LATE-NC stages and neuronal density in the CA1 area. All variables were standardized. The model’s parameters were estimated using the maximum likelihood method, employing the standard NLMINB optimization technique. To verify the model’s accuracy, we evaluated its fit using both relative indices (comparative fit index and Tucker–Lewis index) and absolute indices (standardized root mean square residual and root mean square error of approximation).

## Results

### Association between amygdala-predominant αSyn pathology and hippocampal CA1 subfield neuronal loss

**Figure 1:**
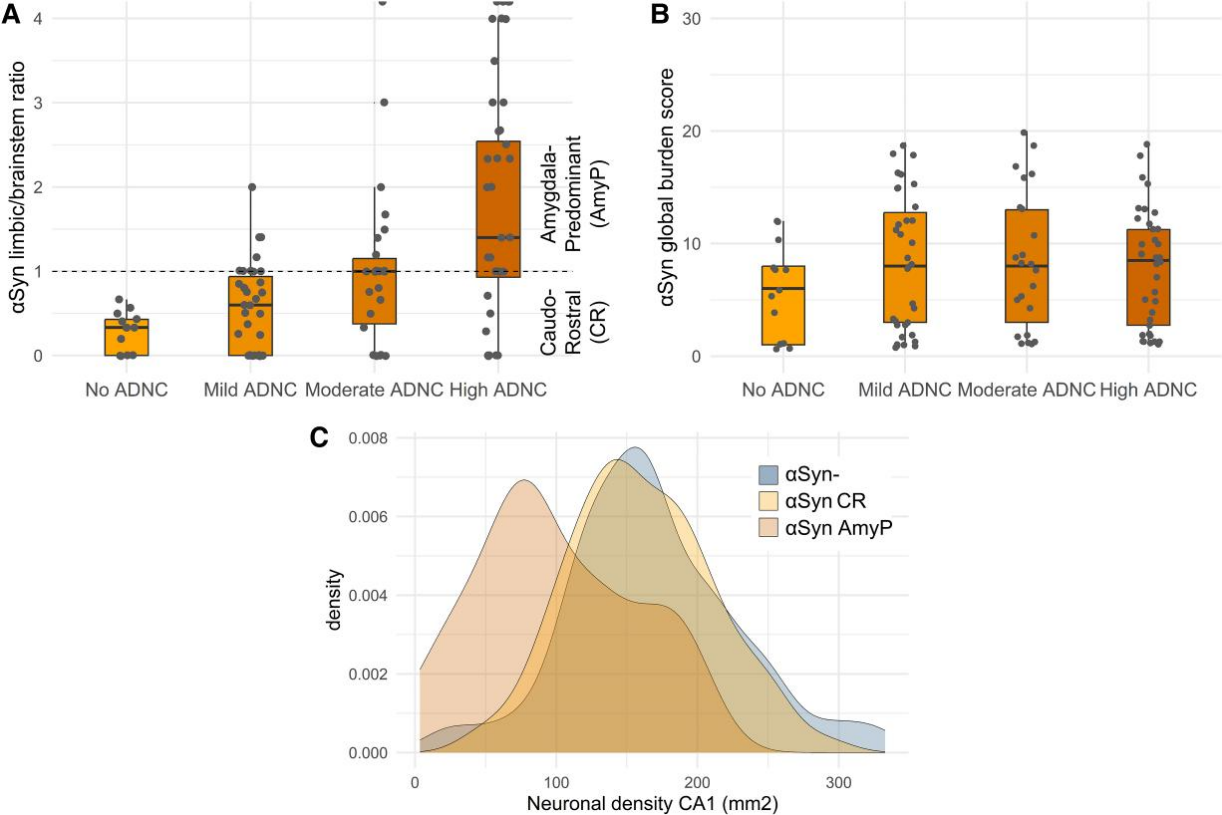
**Two αSyn spreading variants and CA1 neuronal loss.** Boxplots with scattered points (*n* = 107) showing the positive relationship between ADNC severity and the αSyn limbic/brainstem ratio (**A**, Spearman’s *ρ* = 0.51, *P* < 0.001) and the lack of a relationship with the mean αSyn global burden score (**B**, Spearman’s *ρ* = 0.03, *P* = 0.737). Cases with higher pathology severity in the MTL than in the brainstem (ratio > 1) were classified as having an amygdala-predominant pattern (αSynAmyP), while other αSyn pathology exhibiting cases were classified as caudo-rostral (αSynCR). Ratio values that were set to infinity due to no pathology in the brainstem are indicated as half-dots. The horizontal bold lines represent median values, the box margins illustrate the 25 and 75% quartiles, and the whiskers display the lower and upper range values. (**C**) The distribution of neuronal density per 1 mm² in the CA1 region of the posterior hippocampus is shown for αSyn-negative cases (αSyn−, *n* = 179), cases with αSynAmyP (*n* = 34) and αSynCR (*n* = 79). The distributions differ significantly, as confirmed by the Kruskal–Wallis test [χ²(2) = 29.7, *P* < 0.001]. *Post hoc* tests showed that the αSynAmyP distribution is lower than both αSynCR (*P* < 0.001) and αSyn− cases (*P* < 0.001).

We assessed the αSyn pathology severity in seven brain regions from 112 cases exhibiting varying levels of αSyn (Braak LBD stages 1–6). We imputed values from 14 cases with a single missing data point (2.2% of all values). Five cases had more than one missing value and were therefore excluded from further analyses. The distribution of obtained scores for each region is given in Supplementary Table 6. For these αSyn-positive cases, we calculated the αSyn limbic/brainstem ratio and αSyn pathology global burden score. Cases with an αSyn limbic/brainstem ratio > 1 were classified as amygdala-predominant (αSynAmyP, *n* = 34), while the remaining αSyn pathology exhibiting cases were classified as caudo-rostral (αSynCR, *n* = 73) (Fig. 1A). The other cases in our study were labelled αSyn-negative (αSyn-, *n* = 179). The semi-partial Spearman correlation, controlling for age at death, revealed a significant trend of increasing αSyn limbic/brainstem ratio with higher ADNC severity (Fig. 1A, *ρ* = 0.51, *P* < 0.001). However, the overall severity of αSyn pathology, as indicated by the global burden score, did not correlate with an increasing degree of ADNC (Fig. 1B, *ρ* = 0.03, *P* = 0.737).

We observed differences in CA1 neuronal density distribution among cases without αSyn pathology (*n* = 179) and those with two patterns of αSyn pathology using Kruskal–Wallis test (*n* = 107, χ²(2) = 29.69, *P* < 0.001, Fig. 1C). *Post hoc* tests showed that αSynAmyP cases had the lowest CA1 neuronal density [*mean(SD)* = 100.3(55.9)], significantly differing from αSyn− [*mean(SD)* = 167.6(60), *P* < 0.001] and αSynCR [*mean(SD)* = 160(50.2), *P* < 0.001]. There was no significant difference between the αSyn− and αSynCR (*P* = 0.44).

To confirm that the observed effect is not due to other neuropathological changes, we developed a multiple regression model (*n* = 284, Table 1) with αSyn pathology patterns, Braak NFT stages, AβMTL phases, presence of pTDP-43 lesions in the posterior MTL, age at death and sex. Our analysis demonstrated that αSynAmyP was a strong predictor of neuronal density in the CA1 region (*β* = −36, *P* = 0.002) and that this association remained significant even after accounting for Braak NFT stages (*β* = −6.5, *P* = 0.010) and pTDP-43 positivity in the posterior MTL (*β* = −28.9, *P* = 0.002). In contrast, αSynCR did not show a significant negative association with CA1 neuronal density (*β* = −4.6, *P* = 0.543). Similarly, the negative association between AβMTL phases and neuronal density was not significant (*β* = −4.6, *P* = 0.124).

**Table 1:** Results of multiple linear regression investigating the effect of two αSyn pathology patterns on CA1 neuronal loss

	Coef (95% CI)	SE	*t*-value	*P*-value
(Intercept)	165.0 (111.7–218.3)	27.1	6.1	3.72e−09
Age at death	0.4 (−0.3–1.1)	0.4	1.1	0.282
Sex (male)	6.7 (−6.3–19.7]	6.6	1	0.311
Braak NFT stage	−6.5 (−11.5–1.5)	2.5	−2.6	**0**.**010**
Aβ MTL phase	−4.6 (−10.5–1.3)	3	−1.5	0.124
pTDP-43 in post. MTL	−28.9 (−47.1–10.6)	9.3	−3.1	**0**.**002**
αSyn AmyP	−36 (−58.1–13.8)	11.2	−3.2	**0**.**002**
αSyn CR	−4.6 (−19.5–10.3)	7.5	−0.6	0.543

*n* = 284, *R*^2^ = 0.251, *R*^2^ adjusted = 0.232, *P-*value model = 1.13e−14, *F*(7, 276) = 13.2; *P-values* < 0.05 are indicated with bold.

Coef, β coefficient; CI, confidence intervals; SE, standard error; post., posterior; AmyP, amygdala-predominant pattern; CR, caudo-rostral pattern.

To exclude other potential factors impacting neuronal density in our sample, we developed a series of linear models with CA1 neuronal density as the dependent variable and age at death, sex and several potential confounders (including the presence of AGD, ARTAG, the severity of SVD in the temporal lobe, infarctions, seizure disorder, post-mortem interval (PMI) and which hemisphere was used) as independent variables (Supplementary Table 7). We observed that none of these variables affected the relationship between αSynAmyP and CA1 neuronal density. Additionally, we found no significant association between CA1 neuronal density and hemisphere used (*β* = −12.3, *P* = 0.191), AGD (*β* = −1.5, *P* = 0.896), ARTAG (*β* = −7.5, *P* = 0.437) or presence of infarction (*β* = −2.4, *P* = 0.746). As expected, epilepsy was negatively associated with CA1 neuronal density (*β* = −43.9, *P* = 0.004). We also found that the severity of SVD (mainly related to arteriolosclerosis) in the temporal cortex (*β* = −13.4, *P* = 0.001) and PMI (*β* = 0.3, *P* = 0.015) was negatively associated with neuronal density in CA1 but independent of αSyn pathology (Supplementary Table 7).

### The severity of αSyn pathology in amygdala, but not CA1, correlates with CA1 neuronal density

**Figure 2:**
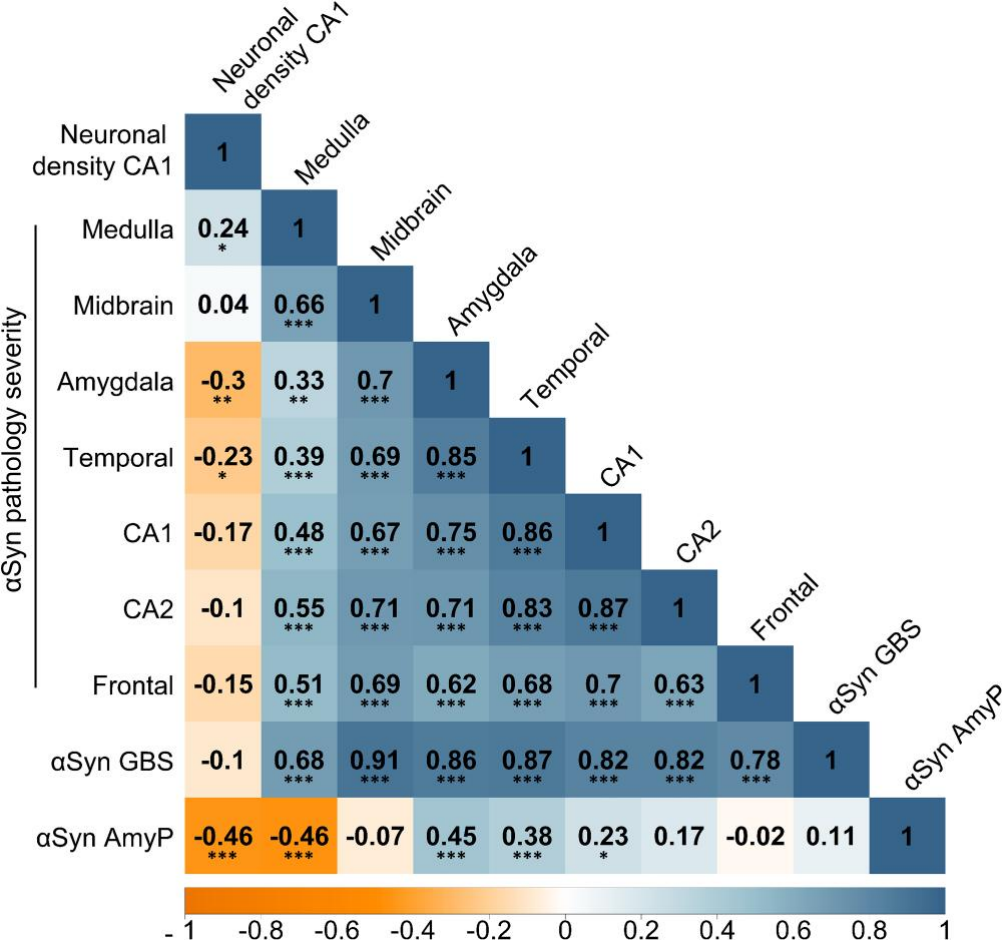
**Association between αSyn pathology severity in assessed brain regions and hippocampal damage.** This figure illustrates the results of Spearman’s semi-partial correlation analysis assessing the relationship between neuronal density in the hippocampal CA1 region and various measures of αSyn pathology, with age at death as a covariate (*n* = 107). The investigated parameters include αSyn pathology severity in different brain regions, amygdala-predominant pattern (αSynAmyP) and αSyn global burden score (αSyn GBS). Each cell in the matrix displays correlation coefficient (*ρ*) representing the strength and direction of the correlation between the corresponding pairs of values. Medulla, medulla oblongata; temporal, temporal cortex; frontal, frontal cortex. *P*-values were adjusted using the Benjamini–Hochberg method. *P < 0.05; **P < 0.01; ***P < 0.001.

To investigate whether the effect of αSyn pathology on CA1 degeneration resulted from its general presence in the brain or its intensity in particular areas, we conducted a semi-partial Spearman correlation analysis on αSyn-positive cases (*n* = 107). αSyn pathology severity scores in various brain regions were considered the predictor variables, neuronal density in the CA1 region the outcome variable, with age at death as a covariate. The results of these correlations are presented in Fig. 2, and the associated corrected *P*-values are detailed in Supplementary Table 8.

This analysis confirmed that neuronal density of the hippocampal CA1 region is negatively associated with the severity of αSyn pathology in the amygdala (*ρ* = −0.3, *P* = 0.003) and posterior temporal cortex (*ρ* = −0.23*, P* = 0.028). Interestingly, no significant relation between CA1 neuronal density and severity of αSyn pathology in CA1 was observed (*ρ* = −0.16*, P* = 0.125) nor in CA2 (*ρ* = −0.1, *P* = 0.371), frontal cortex (*ρ* = −0.15*, P* = 0.160) or substantia nigra in the midbrain (*ρ* = 0.04, *P* = 0.825). A small positive effect was observed for αSyn pathology in the dorsal nucleus of the vagus nerve in the medulla oblongata (*ρ* = 0.24*, P* = 0.021) likely due to its high inverse correlation with the αSynAmyP (*ρ* = −0.46*, P* < 0.001).

Furthermore, all severity scores for αSyn pathology exhibited positive correlations. The strongest relation was observed between the amygdala and temporal cortex (*ρ* = 0.85, *P* < 0.001), CA1 and temporal cortex (*ρ* = 0.86, *P* < 0.001) and between CA1 and CA2 αSyn severities (*ρ* = 0.87, *P* < 0.001). The weakest correlation was seen between the amygdala and medulla oblongata αSyn pathology severity (*ρ* = 0.33, *P* = 0.001). Despite these strong associations between αSyn pathology severities for different regions, the αSyn global burden score did not correlate with decreased CA1 neuronal density (*ρ* = −0.1, *P* = 0.378).

### Different neuropathological profiles in Alzheimer’s cases with amygdala-predominant and caudo-rostral αSyn patterns

**Figure 3:**
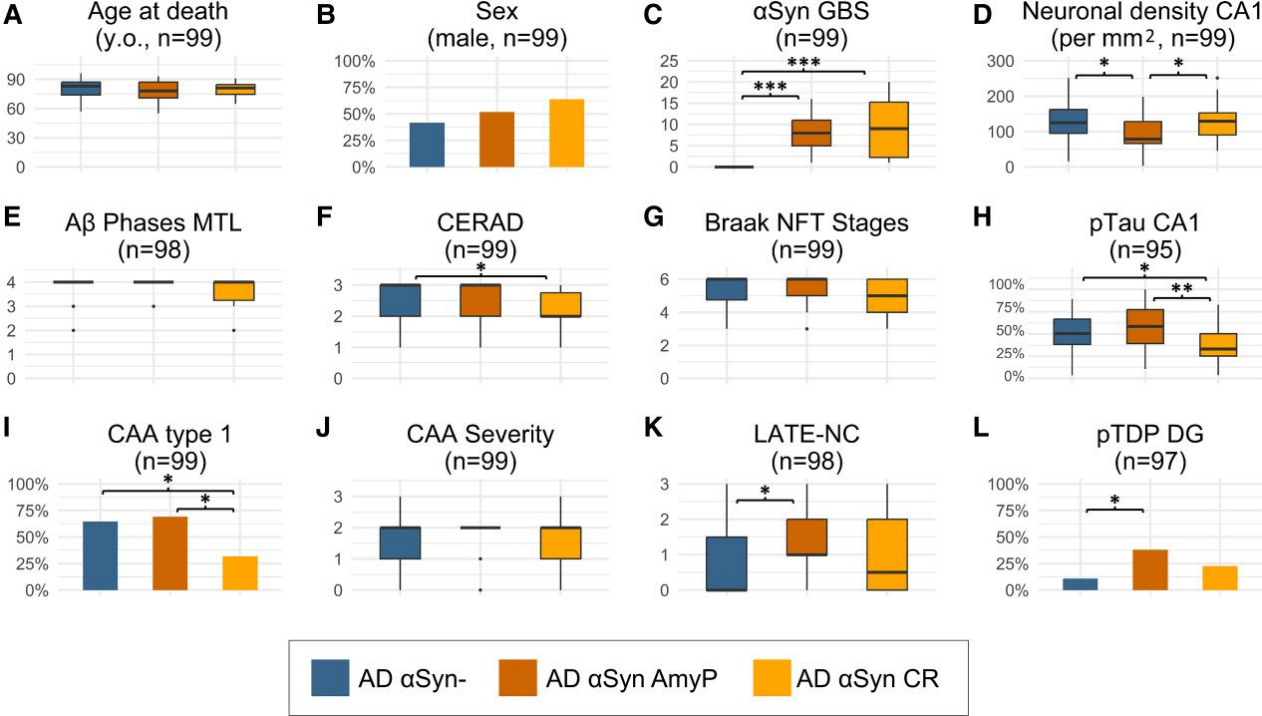
**Characteristics of Alzheimer’s disease patients with and without αSyn pathology**. Alzheimer’s disease patients have been stratified based on the presence and distribution of αSyn pathology into an αSyn-negative group (AD^αSyn−^) and two αSyn-positive groups with amygdala-predominant (AD^αSynAmyP^) or caudo-rostral variants (AD^αSynCR^). The *y*-axis label together with a sample size is indicated above each graph. (**A, C–G, J, K**) Boxplots with horizontal bold lines indicate median values, the box margins represent the 25 and 75% quartiles, while the whiskers show the lower and upper range values. The statistical tests used and their obtained values for numerical variables are given in Supplementary Table 10, while the results of the *post hoc* tests are presented in Supplementary Table 11. (**B, H, I, L**) Bar plots for binary variables show the percentage of cases positive for the demographic or neuropathological feature. The results of two-sided pairwise Fisher’s exact tests are given in Supplementary Table 12. DG, dentate gyrus. *P*-values were adjusted per variable using the Benjamini–Hochberg method. *P < 0.05; **P < 0.01; ***P < 0.001.

Next, we sought to investigate whether Alzheimer’s disease cases with amygdala-predominant and caudo-rostral αSyn pathology patterns differ in their spectrum of associated pathologies (Fig. 3). For this analysis, we selected 99 cases of our cohort with clinically confirmed dementia and moderate/severe ADNC. Within the selected Alzheimer’s disease cases, 48 had no αSyn pathology (AD^αSyn−^), 29 exhibited the amygdala-predominant variant (AD^αSynAmyP^), and 22 showed the caudo-rostral pattern (AD^αSynCR^). Descriptive statistics and the number of cases for each parameter are given in Supplementary Table 9, and the applied statistical tests and their results are given in Supplementary Tables 10–12.

The three above-mentioned groups did not differ in their age at death [Fig. 3A, *F(2,96)* = 0.66, *P* = 0.518] and sex (Fig. 3B, *P* = 0.230). Additionally, both AD^αSynAmyP^ and AD^αSynCR^ had the same levels of αSyn global burden score (Fig. 3C, *Z* = −0.16, *P* = 0.875). AD^αSynAmyP^ had a lower CA1 neuronal density compared with AD^αSyn−^ (*Z* = −2.5, *P* = 0.037) or AD^αSynCR^ (*Z* = −2.19, *P* = 0.043) (Fig. 3D).

While AD^αSynAmyP^ displayed comparable levels of ADNC to AD^αSyn−^, AD^αSynCR^ generally exhibited a lower degree of ADNC severity (Fig. 3E–H). Statistical analysis revealed that AD^αSynCR^ differed significantly from the AD^αSyn−^ group in the severity of neuritic plaques, reflected by the CERAD score (*Z* = 2.66, *P* = 0.023), and that they had fewer pTau-positive neurons in CA1 than the other groups (*Z* = 2.24, *P* = 0.038; *Z* = 3.16, *P* = 0.005). Interestingly, AD^αSynCR^ had the lowest rate of developing CAA type 1, i.e. CAA with capillary involvement (Fig. 3I, *P* = 0.026), although there was no difference in CAA severity among groups (Fig. 3J, *H(2)* = 2.1, *P* = 0.351). On the other hand, AD^αSynAmyP^ had the same prevalence of CAA type 1 as AD^αSyn−^ (*P* = 0.81). However, AD^αSynAmyP^ had significantly higher LATE-NC stages (Fig. 3K, *Z* = 2.63, *P* = 0.03) and showed more prevalent pTDP-43 inclusions in the dentate gyrus (Fig. 3L, *P* = 0.02) than AD^αSyn−^.

### Colocalization of α-synuclein, pTDP-43 and pTau in Alzheimer’s disease and their synergistic effect on CA1 neuron loss

To better understand the relations between Alzheimer’s disease-related neuropathologies in the MTL and CA1 degeneration, we investigated potential mediatory effects using a path analysis model. This model used 95 out of 99 Alzheimer’s disease cases, excluding the four cases with missing values for at least one neuropathological parameter. The model’s directionality was informed by the literature indicating that TDP-43 and tau pathologies contribute to neuronal death through mechanisms such as loss of normal protein function, impaired neuronal transport^80,81^ and induction of regulated cell death^82,83^ and by research demonstrating that αSyn can induce tau and TDP-43 aggregation in animal and cell models.^53,77-79^ It estimated eight model parameters, including five regressions between neuropathological variables and three exogenous variances (Supplementary Fig. 3). The model demonstrated a good fit based on both relative and absolute fit measures, adhering to standard guidelines (Supplementary Table 13).^84^

**Figure 4:**
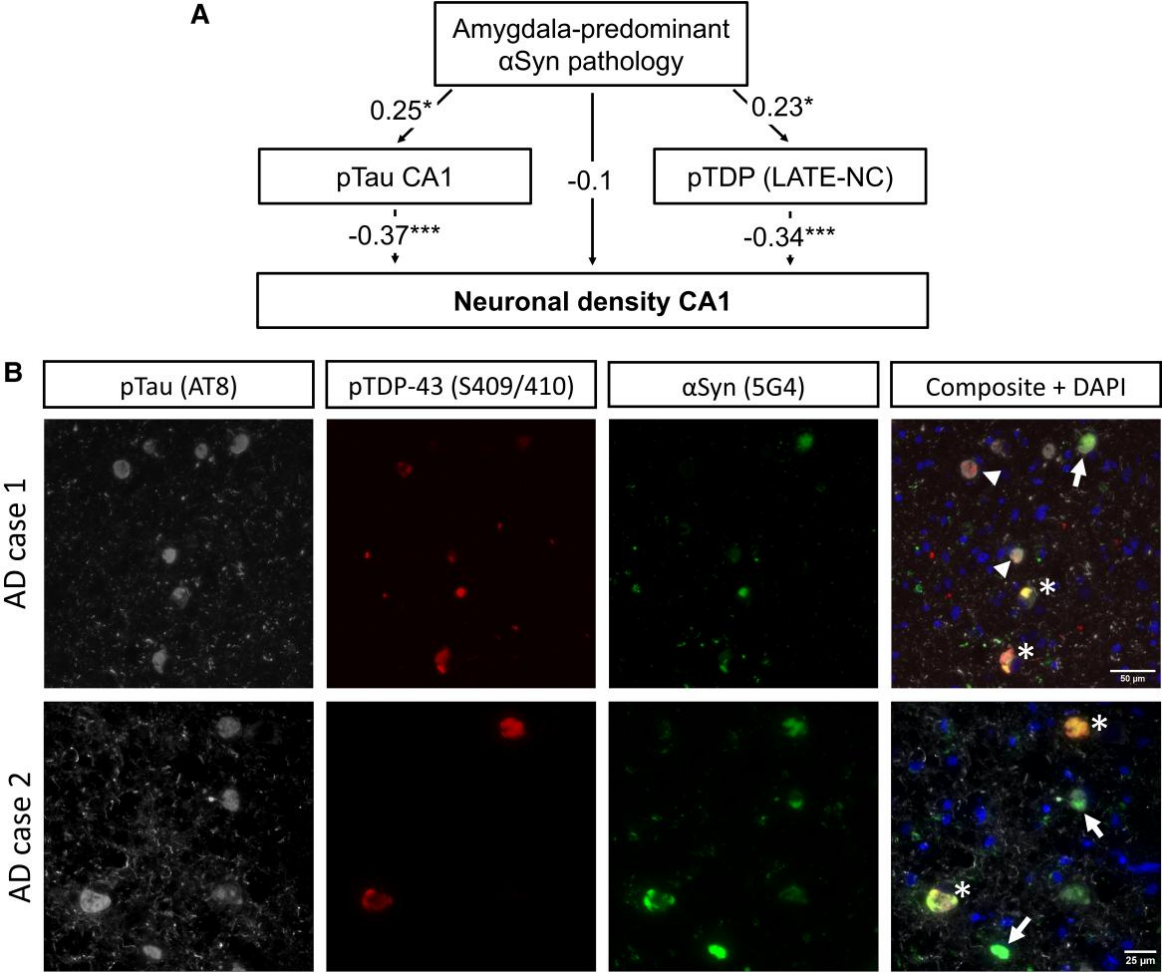
**The interactions between Alzheimer’s disease-related neuropathological pathologies and decreased CA1 neuronal density.** (**A**) The graphical representation of the path analysis model was designed to test whether amygdala-predominant pattern (αSynAmyP) has a direct association with neuronal density in CA1 or whether it is mediated by other pathologies in Alzheimer’s disease patients (*n* = 95). The observed variables are represented by square boxes and include the presence of αSynAmyP, the percentage of neurons affected by pTau in CA1, stages of LATE-NC and neuronal density in the CA1 region. Arrows represent regression paths connecting the variables, with the direction of the arrow indicating the flow from the independent to the dependent variable. These arrows are annotated with standardized regression coefficients (estimates). Detailed results of the model are given in Supplementary Table 13. *P < 0.05; **P < 0.01; ***P < 0.001. (**B**) Triple immunofluorescence staining of the amygdala from two Alzheimer’s disease cases exhibiting LATE-NC and amygdala-predominant αSyn co-pathologies. The specific antibodies used are indicated above each panel. Asterisks (*) mark inclusions positive for all three markers. Arrows indicate aggregates positive for both pTau and pTDP-43, while arrowheads denote aggregates positive for pTau and αSyn. The scale bar in the composite image applies for all images of a given case.

Based on the model results (Fig. 4A), we observed that αSynAmyP was related to both an increase in pTau pathology density in CA1 [*std. estimate(SE)* = 0.25 (0.1), *P* = 0.013] and LATE-NC [*std. estimate(SE)* = 0.23 (0.1), *P* = 0.022]. We also observed the direct significant effect of pTau [*std. estimate(SE)* = −0.37 (0.09), *P* < 0.001] and LATE-NC [*std. estimate(SE)* = 0.34 (0.09), *P* < 0.001] on CA1 neuronal density. However, αSynAmyP had a non-significant direct relationship with CA1 neuronal density [*std. estimate(SE)* = −0.1 (0.09), *P* = 0.287] only an indirect one via pTau and TDP-43.

Triple staining for pTDP-43, aggregated αSyn and pTau was performed on five clinically demented Alzheimer’s disease cases with LATE-NC and αSynAmyP. In four out of five cases, we observed partial colocalization of pTDP-43, aggregated αSyn and pTau-positive deposits in the amygdala (Fig. 4B). Similar triple-positive lesions were also identified in the posterior MTL, including the hippocampus and/or temporal cortex, in these four cases. The single case that was negative for triple staining in the MTL exhibited a low burden of αSyn pathology, with moderate involvement in the amygdala and no pathology in the posterior hippocampus and temporal cortex.

## Discussion

In this study, we demonstrated an association between amygdala-predominant αSyn pathology and neuronal loss in the hippocampal CA1 region. This association persisted even after accounting for potential confounders, including overall ADNC severity, hippocampal pTDP-43, SVD, AGD, ARTAG, PMI, infarctions and seizure disorder. We found that the severity of αSyn pathology in the amygdala, but not in CA1, correlated with CA1 neuron loss. Additionally, the path analysis model showed that in Alzheimer’s disease cases, the relationship between amygdala-predominant αSyn and CA1 degeneration is partially mediated by the limbic burden of pTau and LATE-NC, indicating a complex effect beyond the previously reported neurotoxicity of αSyn aggregates.^85,86^ Interestingly, Alzheimer’s disease cases with caudo-rostral αSyn pathology did not exhibit CA1 neuronal loss, suggesting that different αSyn spreading patterns may be linked to specific neuropathological profiles and could have potential clinical implications.

The amygdala-predominant αSyn subtype was previously considered benign due to a lack of clear association with cognitive impairment^7,87^ and was traditionally categorized by mild pathology confined to the amygdala.^11^ However, recent developments in neuropathological subtyping suggest that cases should be grouped based on their spreading patterns rather than mere regional involvement.^12-14,88^ Here, we distinguished caudo-rostral cases from amygdala-predominant ones using the αSyn limbic/brainstem severity ratio. This stratification revealed that the amygdala-predominant αSyn variant is strongly associated with CA1 neuronal loss, whereas the caudo-rostral αSyn pathology pattern is not. Comparing neuropathological characteristics, cases with the amygdala-predominant type had more severe LATE-NC, more common pTDP-43 inclusions in the dentate gyrus and a greater burden of pTau in CA1. These findings support study showing a high prevalence of amygdala αSyn pathology and LATE-NC in the limbic-predominant Alzheimer’s disease subtype,^3^ suggesting pronounced damage to limbic tissue in these patients.

The amygdala, with its early involvement in all Alzheimer’s disease-related pathologies, is considered an important region in neurodegenerative diseases.^89^ Anatomical connections between the amygdala and the CA1 subfield of the hippocampus have been demonstrated in tracer studies.^30-32^ This research highlighted the important role of the basal and accessory basal nuclei, which have both efferent and afferent connections with CA1.^30-32^ Given these direct connections, the role of amygdala in hippocampal neurodegeneration could be attributed to several mechanisms, including providing a physical space for interactions among misfolded proteins, facilitating transsynaptic spread of aggregates such as pTau or αSyn towards hippocampus or by causing synaptic damage due to death of connected neurons.

The path model presented in this study shows that amygdala-predominant αSyn pathology is related to both pTau and pTDP-43 limbic pathology in Alzheimer’s disease. Furthermore, using triple fluorescence staining, we found lesions in the hippocampus and amygdala positive for pTau, pTDP-43 and aggregated αSyn in Alzheimer’s disease cases with amygdala-predominant αSyn pathology. Colocalization of pTau and αSyn has been consistently shown in various diseases (Alzheimer’s disease, LBD, multiple system atrophy and Pick’s disease) and different brain areas, with the strongest evidence found in the limbic system.^6,90-94^ Colocalization between TDP-43 and αSyn was detected in the limbic areas of post-mortem tissue^52,95^ and in HeLa cells co-transfected with the prion-like C-terminal domain of TDP-43 and αSyn.^96^ TDP-43 was also shown to colocalize and interact with pTau in human brain tissue.^97^ Previous studies using animal and cell models have demonstrated that αSyn aggregates can induce the fibrillization and aggregation of tau^49,53,78,79^ and TDP-43.^53,77^ Simultaneous overexpression of αSyn and TDP-43 has been shown to increase neurotoxicity.^54^  *In vitro* studies indicate the potential formation of hybrid fibrils between these proteins, amplifying their neurotoxic effects.^96,98,99^ Our triple labelling experiment extends these findings by demonstrating the colocalization of all three aggregating proteins αSyn, pTau and pTDP-43. Taking all this evidence into consideration, we hypothesize that an interplay between αSyn, pTau and pTDP-43 in the limbic system might play an important role in the neurodegenerative process of Alzheimer’s disease by identifying a specific subtype defined by amygdala-predominant αSyn pathology.

Alternatively, αSyn aggregates may independently contribute to increased hippocampal neurodegeneration. Several points support this hypothesis: (i) In LBD, αSyn accumulation typically results in dopaminergic neuron loss in the brainstem through various neurotoxic mechanisms, including autophagy disruption, without involving pTau or pTDP-43.^85,86^ (ii) It has been also shown that small soluble αSyn fibrils, <200 nm in length, could be more toxic than larger aggregates.^100^ Such small fibrils are usually not detected with standard immunohistochemical methods, which would explain why we observed CA1 damage in cases without αSyn immunoreactivity in the hippocampus. (iii) Finally, although we did not detect a direct effect of αSyn on CA1 neuronal density in Alzheimer’s disease samples, our model on the full cohort, including controls, indicated that the overall relationship between amygdala-predominant αSyn pathology and CA1 neuronal density was observable after controlling for hippocampal TDP-43 and Braak NFT stages. This seemingly contradictory result may be explained by the differences in the samples: when comparing Alzheimer’s disease cases, only Alzheimer’s disease-specific differences can be observed, whereas, in the entire sample, non-Alzheimer’s disease-related αSyn lesions may contribute to the neurodegenerative process as well. These findings raise important questions about whether αSyn and TDP-43 contribute to hippocampal damage synergistically or additively, highlighting the need for experimental investigation to resolve this issue.

This study also underscores the impact of other neuropathological lesions on hippocampal damage. Our findings indicate that while SVD in the temporal grey matter does not explain the relationship between amygdala-predominant αSyn pathology and CA1 damage, it was independently associated with a decreasing neuronal count in this region, consistent with previous studies on hippocampal sclerosis.^25,27^ This effect may be partly due to its interaction with LATE-NC,^101,102^ which accelerates neuron loss in CA1,^103^ although the precise mechanisms remain unclear. Furthermore, cellular mechanisms underlying neuron loss in Alzheimer’s disease cases with multiple co-pathologies are not fully understood. Previous research suggests that necroptosis may play a significant role in TDP-43-related cell death,^103^ warranting further investigation.

Defining the distinct neuropathological characteristics of αSyn pathology spreading variants in Alzheimer’s disease patients could contribute to a better understanding of the Alzheimer’s disease spectrum and improved patient stratification in the future. The recent advancements in αSyn seeding assays,^104-107^ such as the real-time quaking-induced conversion assay,^108^ may play an important role in patient-tailored diagnostic approaches. While these assays have been extensively evaluated in cohorts with LBD, their diagnostic accuracy in Alzheimer’s disease patients remains unclear. Studies utilizing these assays have detected αSyn positivity in 30–45% of cases,^109,110^ slightly lower than the neuropathological estimation of 43–63%.^4-7^ This raises the question of whether these biomarkers enable the distinction between specific spreading patterns of αSyn pathology. For clinical purposes, differentiating between Alzheimer’s disease patients with various αSyn patterns is crucial, as possible αSyn interaction with other protein aggregates may impact clinical progression and treatment options for patients. Given the heterogeneity of pathological processes observed in Alzheimer’s disease patients with additional αSyn aggregates, treatment with anti-amyloid monoclonal antibodies^111^ may be less effective compared with other groups. Furthermore, the observation that Alzheimer’s disease patients with severe hippocampal degeneration often exhibit comorbid αSyn and TDP-43 pathologies provides insights that could be utilized to improve diagnostic accuracy for patients *in vivo*, enhancing the reliability of clinical trials by better stratification.

This study has several limitations. Firstly, cases with an amygdala-predominant αSyn pathology pattern in our sample may be underdiagnosed, especially with low lesion burden. This is due to the highly heterogeneous nature of the amygdaloid complex and analysing only a representative section. Additionally, the dual hypothesis of αSyn pathology spread assumes that the amygdala-predominant variant begins unilaterally,^112^ and our study only examined one hemisphere microscopically. Encouragingly, we observed a similar ratio of ∼2-to-1 between cases with caudo-rostral and amygdala-predominant patterns, as Raunio,^13^ with a corresponding increase in amygdala-based αSyn pathology with increasing ADNC severity. Another limitation is our inability to further stratify individuals with the caudo-rostral pattern into those with early versus late olfactory bulb involvement due to the lack of tissue from this region.^15^ Finally, the stringent exclusion and stratification criteria reduced the number of cases per group. While our path analysis model meets the minimum criterion of 10 cases per estimate, it does not reach the optimal standard of 20 cases per parameter estimate,^113^ increasing the likelihood of overlooking minor effects.

## Conclusion

**Figure 5:**
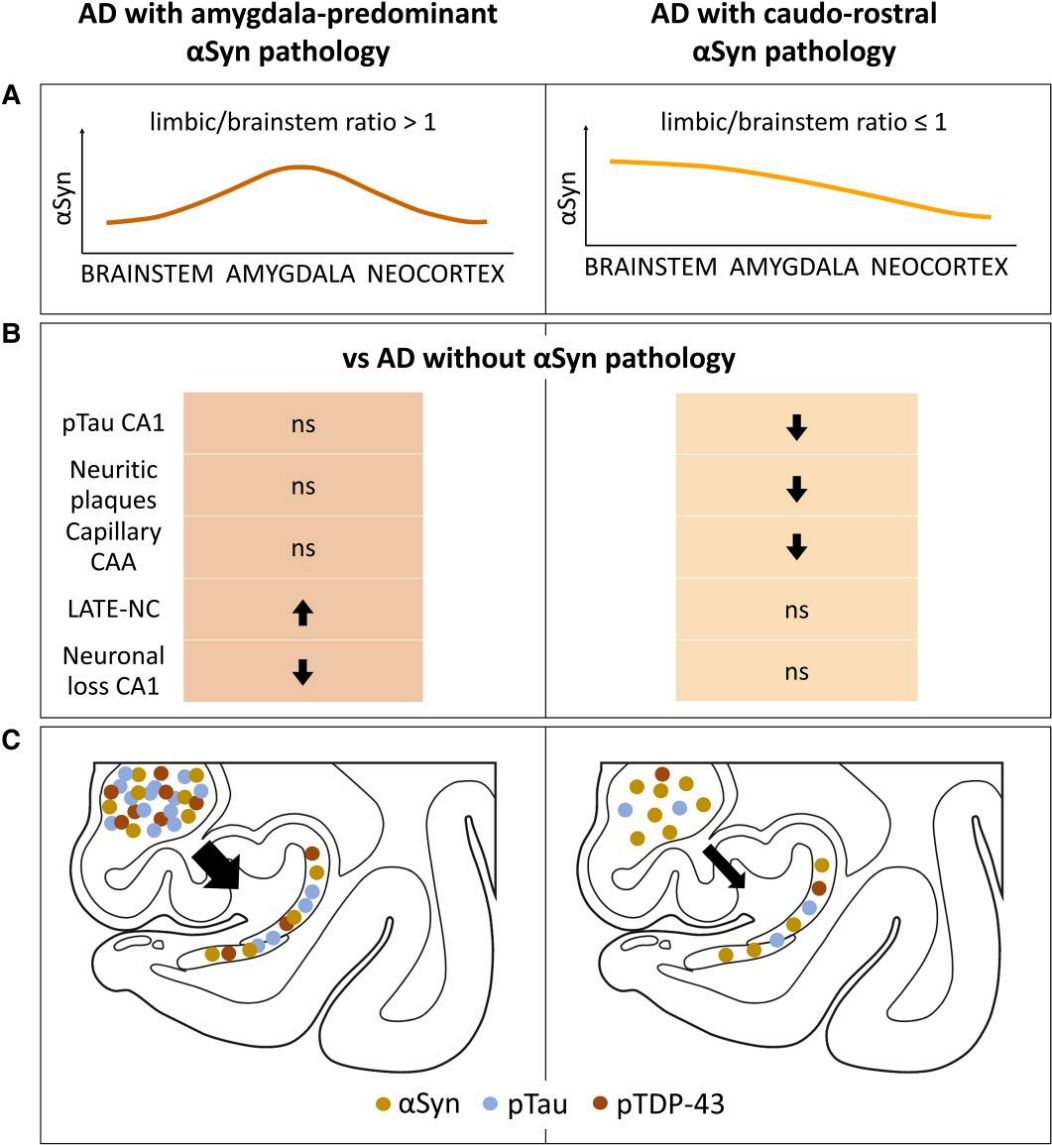
**A summary diagram illustrating the distinctions between Alzheimer’s disease with caudo-rostral and amygdala-predominant αSyn pathology.** (**A**) Depiction of the simplified distribution of αSyn pathology severity for two spreading patterns. (**B**) Neuropathological differences between Alzheimer’s disease with two αSyn pathology spreading patterns to Alzheimer’s disease without αSyn pathology were found in this study. An upward arrow indicates an increase in the parameter for the αSyn group compared with Alzheimer's disease without αSyn pathology, while a downward arrow indicates a decrease compared to the Alzheimer’s disease group. ns, not significant. (**C**) Proposed mechanisms underlying increased hippocampal neuronal loss in Alzheimer’s disease with amygdala-predominant αSyn pathology, where the amygdala acts as a hub for interaction and heightened aggregation of αSyn, pTDP-43 and pTau, resulting in elevated triple pathology in the hippocampus. The interaction might be less pronounced in the caudo-rostral variant.

Our findings indicate that amygdala-predominant αSyn pathology in Alzheimer’s disease is linked to hippocampal degeneration. The precise nature of αSyn contribution to neuronal loss—whether additive or synergistic with other Alzheimer’s disease-related protein aggregates—remains to be clarified. However, the presence of lesions with co-localized pTau, TDP-43 and αSyn, along with path analysis showing that pTau and pTDP-43 partially mediate the relationship between αSyn pathology and CA1 neuronal loss, supports the hypothesis of interactions among these proteins. The distinct neuropathological characteristics observed in Alzheimer’s disease patients with amygdala-predominant versus caudo-rostral αSyn pathology patterns, coupled with the potential interplay among pTau, pTDP-43 and αSyn, highlight the critical need for precise patient stratification (Fig. 5).

## Supplementary Material

fcae442_Supplementary_Data

## Data Availability

Summary statistics of basic demographic, clinical and neuropathological parameters for each group used in this study are given in Supplementary Table 1. Due to legislation and privacy protection, any medical reports and files of the cases included in this study cannot be made available. Pseudonymized data will be provided by the corresponding author upon reasonable request. The R code used in this manuscript is given in the Supplementary Materials.
