# Supplementary material for: Amygdala-predominant α-synuclein pathology is associated with exacerbated hippocampal neuron loss in Alzheimer’s disease
Source: Brain Commun. 2024 Dec 5;6(6):fcae442. doi: 10.1093/braincomms/fcae442 (PMC11631359; doi:10.1093/braincomms/fcae442)
Supplement: fcae442_Supplementary_Data [file fcae442_supplementary_data.pdf]

Amygdala-predominant α-synuclein pathology is associated with exacerbated hippocampal neuron loss in Alzheimer's disease

Klara Gawor, Sandra O. Tomé, Rik Vandenberghe, Philip Van Damme, Mathieu Vandenbulcke, Markus Otto, Christine A.F. von Arnim, Estifanos Ghebremedhin, Alicja Ronisz, Simona Ospitalieri, Matthew Blaschko, and Dietmar R. Thal

Supplementary Material

Supplementary Figure 1 Representative photos of α-synuclein (αSyn) pathology burden for each semi-quantitative severity score

Supplementary Figure 2 Exemplary photos used for neuronal quantification

Supplementary Figure 3 Illustration of the path analysis model employed in this study

Supplementary Table 1 The clinical and neuropathological characteristics of the study cohort

Supplementary Table 2 Exclusion criteria

Supplementary Table 3 Antibodies used in this study

Supplementary Table 4 Parameters used in this study

Supplementary Table 5 Regions with αSyn pathology severity assessment

Supplementary Table 6 Distribution of αSyn pathology severity scores across different brain areas in αSyn-positive cases

Supplementary Table 7 Results of linear regressions testing the effect of confounders on the relations between two patterns of αSyn pathology and CA1 degeneration

Supplementary Table 8 Corrected P-values from Spearman's semi-partial correlation, with age at death as a covariate, for αSyn-positive cases

Supplementary Table 9 Key differences between symptomatic Alzheimer's disease patients without αSyn and with different spreading patterns of αSyn pathology

Supplementary Table 10 Results of ANOVA and Kruskal-Wallis tests on Alzheimer's disease patients

Supplementary Table 11 Results of a post-hoc Dunn's multiple comparisons tests on Alzheimer's disease patients

Supplementary Table 12 Results of pairwise-comparisons with Fisher's exact test on nominal variables on Alzheimer's disease patients

Supplementary Table 13 Results and fit statistics of the path analysis model using observations from Alzheimer's disease patients

Supplementary references

R code

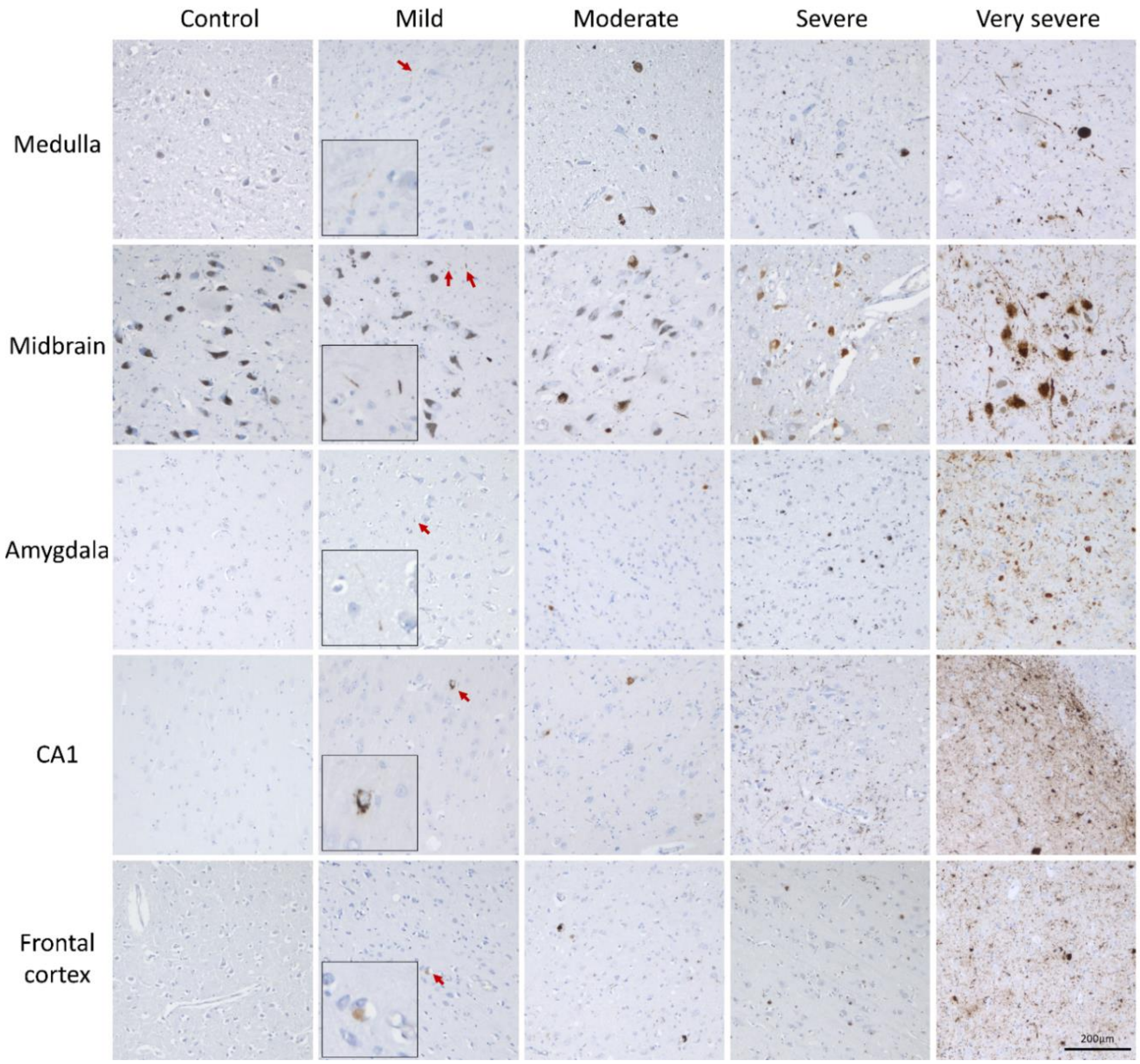

Supplementary Figure 1 Representative photos of αSyn pathology burden for each semi-quantitative severity score. The graphic representation displays αSyn severity scores assigned to various brain regions, including the dorsal vagal nucleus in the medulla, substantia nigra in the midbrain, amygdala, CA1 subregion of the posterior hippocampus, and grey matter in the frontal cortex. Arrows in the mild condition indicate αSyn pathology lesions, further illustrated at higher magnification. The tissue was stained using the 5G4 antibody and photos were taken at a magnification of 200x. Established criteria from the third report from the Dementia with Lewy Bodies Consortium[1] have been used to evaluate the severity of pathology in the amygdala, temporal cortex, and frontal cortex. Given the substantial variability in αSyn lesions across different brain regions, for other regions we used additional criteria. In the medulla oblongata, we defined mild αSyn pathology as the presence of up to three lesions in the vicinity of the vagal nucleus, while a

moderate rating was assigned when more than three lesions were observed, but without involvement in other medullar areas. A severe score was assigned when numerous lesions were visible in the vagal nucleus, with sparse lesions outside this region. A very severe score denoted almost complete αSyn involvement in the vagal nucleus and numerous inclusions in other areas. For the midbrain, our assessment focused solely on the substantia nigra. A mild pathology was characterized by fewer than three lesions, moderate pathology exhibited more than three lesions but with a still sparse distribution, severe pathology had numerous lesions, and very severe pathology indicated that more than half of the melanin-rich cells were affected by αSyn pathology. In the subfields CA1/CA2 of the hippocampus, mild pathology was defined as having fewer than three lesions, moderate pathology entailed more than three lesions but with a still sparse distribution, severe pathology comprised numerous lesions, and very severe pathology was distinguished by involvement that was visually evident even at low magnification.

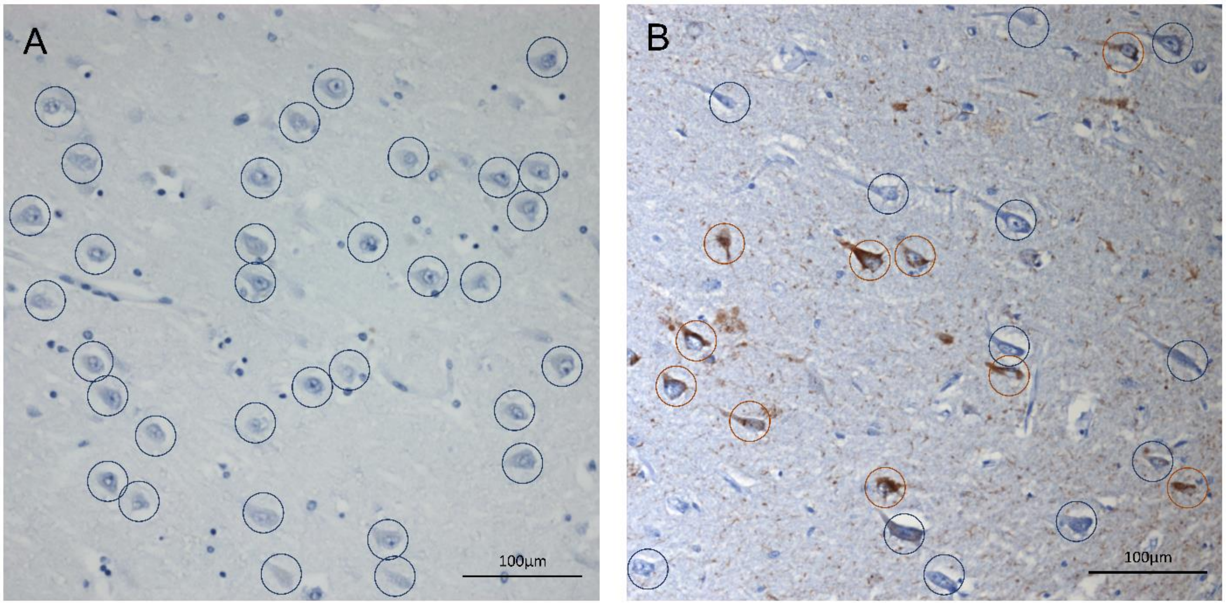

Supplementary Figure 2 Exemplary photos used for neuronal quantification. A. A photo of CA1 subfield tissue stained with an antibody against pTDP-43 (409/410) and counterstained with hematoxylin (magnification x200), with manual annotations of neurons (blue circles). Three such photos were used to quantify the density per mm^2. **B.** A photo of CA1 subfield tissue stained with an antibody against pTau (AT8) and counterstained with hematoxylin (magnification x200). pTau-positive neurons (red circles) and pTau-negative neurons (blue circles) were counted in three such photos, and the percentage of affected neurons was quantified.

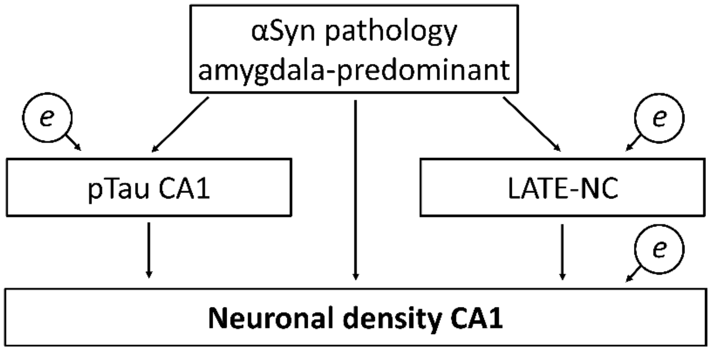

Supplementary Figure 3 Illustration of the path analysis model employed in this study The exogenous observed variable is amygdala-predominant αSyn pathology, while the endogenous observed variables include pTau in CA1, LATE-NC, and neuronal density in CA1. For each endogenous variable, the residual error term (depicted as small circles) has been estimated to account for unexplained variance. Arrows denote regression paths linking the variables, indicating the direction of influence from the independent to the dependent variable.

Supplementary Table 1 The clinical and neuropathological characteristics of the study cohort (*n* = 291)

		αSyn pathology negative				αSyn pathology positive			
		No ADNC (*n* = 45)	Mild ADNC (*n* = 69)	Moderate ADNC (*n* = 31)	Severe ADNC (*n* = 34)	No ADNC (*n* = 13)	Mild ADNC (*n* = 38)	Moderate ADNC (*n* = 23)	Severe ADNC (*n* = 38)
Mean (SD)	Age at death	69.7 (9.7)	73.8 (9)	80.3 (10)	79.5 (9.9)	70.9 (7.2)	76.7 (9.5)	82.9 (8.2)	77.1 (8.7)
	Braak NFT Stages	1.1 (1)	1.7 (0.8)	3.8 (0.7)	5.7 (0.4)	1.7 (1.2)	1.8 (0.8)	3.7 (0.8)	5.6 (0.5)
	Aβ Phases MTL	0 (0)	1.8 (0.9)	3.5 (0.7)	3.9 (0.3)	0 (0)	2.1 (1.2)	3.5 (0.6)	4 (0.2)
	CERAD	0 (0)	0.1 (0.3)	1.5 (0.9)	2.8 (0.4)	0 (0)	0.4 (0.8)	1.5 (0.7)	2.5 (0.5)
	Neuronal density CA1	183.1 (54.9)	182.8 (53.7)	156.5 (61.9)	126.6 (57.5)	158.1 (29.8)	161.7 (53)	150.4 (63.3)	100.4 (55.1)
	Braak LBD Stages	0 (0)	0 (0)	0 (0)	0 (0)	3.2 (1.8)	4 (1.9)	4.2 (1.8)	4.4 (1.7)
	PMI (*n*=268)	41 (23.5)	45.9 (32.7)	31 (23.6)	17.4 (14.7)	48.6 (38.3)	33.9 (26.5)	20.5 (13.5)	20.3 (24.3)
	SVD temporal (*n*=240)	0.8 (1)	0.8 (0.8)	0.8 (0.9)	1.2 (0.7)	1.1 (0.8)	1.3 (0.9)	0.8 (0.7)	1.5 (0.9)
% of cases	αSyn AmyP (*n*=286)	0%	0%	0%	0%	0%	11%	30%	61%
	Sex (Male)	60%	48%	42%	44%	77%	66%	43%	58%
	pTDP43 hipp.	2%	7%	16%	29%	0%	11%	57%	39%
	Epilepsy	4%	1%	6%	18%	8%	5%	4%	3%
	Dementia	0%	4%	48%	97%	15%	37%	70%	97%
	AGD (*n*=269)	18%	10%	18%	0%	15%	19%	0%	3%
	ARTAG (*n*=158)	22%	30%	5%	22%	0%	18%	17%	22%
	Infarcts (*n*=273)	38%	31%	38%	35%	31%	41%	38%	24%
	Hemisphere (left)(*n*=286)	7%	4%	6%	47%	0%	24%	26%	18%

The mean and standard deviations (SD) of factors regarded as continuous variables are in the upper panel whereas nominal variables are summarized by % of cases with such characteristics. If a specific parameter has not been analyzed for the entire cohort, the sample size (n) is provided in brackets.

ADNC – Alzheimer's disease neuropathological changes according to Montine et al. 2012[1]. AGD – argyrophilic grain disease. αSyn AmyP – amygdala-predominant αSyn pathology. ARTAG – aging-related Tau astrogliopathy. CERAD – consortium to establish a registry of Alzheimer's disease. MTL – medial temporal lobe. LBD – Lewy body disease. NFT – neurofibrillary tangle. PMI – Post-mortem interval. SVD – Small vessel disease.

Supplementary Table 2 Exclusion criteria

- Age of death <50y.o
- Presence of tumors or cancers in the central nervous system
- Central nervous system infections
- Severe brain edema or hypoxia
- Diagnosis of neurodegenerative conditions (multiple sclerosis, Huntington's disease, frontotemporal lobar degeneration, multiple system atrophy)

Supplementary Table 3 Antibodies used in this study

	Antibody name	Source	Clonality; Host	Dilution	Stained regions
Aβ	Aβ17-24 (4G8)	BioLegend, US	Monoclonal; Mouse	1:5000	anterior MTL, occipital cortex
pTau	pTau Ser202, Thr205 (AT8)	Thermo Fisher, US	Monoclonal; Mouse	1:1000	anterior MTL, posterior MTL, occipital cortex
αSyn	Anti-Aggregated αSyn (5G4)	Merck Millipore, Germany	Monoclonal; Mouse	1:2000	medulla oblongata, anterior MTL (if positive: brainstem, pons, posterior MTL, frontal cortex)
pTDP-43	pTDP-43 409/410	Cosmobio Co. LTD, Japan	Polyclonal, Rabbit	1:5000	anterior MTL, posterior MTL (if positive: frontal cortex)

Supplementary Table 4 Parameters used in this study

Staining	Parameter	Operationalization	Values
Aβ	AβMTL phases	Phases of Aβ plaque distribution in the MTL (Standard guidelines)	0-4
	CAA Severity	Severity of CAA (Standard guidelines)	0-3
	CAA Type 1	Presence of CAA with capillary involvement	0/1
pTau	Braak NFT stages	Spread of pTau pathology (Standard guidelines)	0-6
	pTau density in CA1	The proportion of pTau-positive neurons in CA1	0-100%
	ARTAG	Presence of ARTAG (Standard guidelines)	0/1
	AGD	Presence of AGD (Standard guidelines)	0/1
	CERAD score	Severity of neuritic plaques (Standard guidelines)	0-3
pTDP-43	LATE-NC stages	Severity of LATE-NC (Standard guidelines)	0-3
	pTDP in DG	Presence of pTDP-43 inclusions in dentate gyrus of posterior hippocampus	0/1
	pTDP in post. MTL	Presence of pTDP-43 inclusions in posterior hippocampus and temporal lobe	
	Neuronal density CA1	Manually quantification of neuronal density per $1mm^2$ in CA1 subfield of posterior hippocampus	0-Inf
αSyn	Severity	Semiquantitative severity score of αSyn burden in 7 brain regions	0-4
	Global burden score (GBS)	The sum of αSyn severity scores across five brain regions (medulla, midbrain, amygdala, temporal cortex, frontal cortex)	0-20
	Limbic/brainstem ratio	Amygdala+Temporal cortex/Medulla+Midbrain (αSyn severity)	0-Inf
	Braak LBD stages	Progression of aSyn pathology (Standard guidelines)	0-6
Hematoxylin-eosin	SVD	Semiquantitative severity of temporal lobe arteriolosclerosis	0-3

AGD – argyrophilic grain disease. ARTAG – aging-related Tau astrogliopathy. CAA – cerebral amyloid angiopathy. CERAD – consortium to establish a registry of Alzheimer's disease. DG – dentate gyrus. LATE-NC – limbic-predominant age-related TDP-43 encephalopathy neuropathologic change. LBD – Lewy body disease. MTL – medial temporal lobe. NFT – neurofibrillary tangle. pTau – phosphorylated Tau. SVD – small vessels disease.

Supplementary Table 5 Regions with αSyn pathology severity assessment

			Application			
	**Region analyzed**	**Tissue block**	**Braak staging**	**Severity assessment**	**Global severity score**	**Limbic/brainstem ratio**
Medulla	Dorsal vagal nucleus	Medulla oblongata	☑	☑	☑	☑
Pons	Locus coeruleus	Pons	☑	☒	☒	☒
Midbrain	Substantia nigra	Midbrain	☑	☑	☑	☑
Amygdala	Amygdaloid complex	Anterior MTL	☑	☑	☑	☑
CA1	CA1 subfield of hippocampus	Posterior MTL	☒	☑	☒	☒
CA2	CA1 subfield of hippocampus	Posterior MTL	☒	☑	☒	☒
Temporal	Parahippocampal gyrus	Posterior MTL	☑	☑	☑	☑
Frontal	Middle frontal gyrus	Middle frontal gyrus	☑	☑	☑	☒

MTL – medial temporal lobe.

Supplementary Table 6 Distribution of αSyn pathology severity scores across different brain areas in αSyn-positive cases (*n* = 107)

	Medulla	Midbrain	Amygdala	Temporal	CA1	CA2	Frontal
No αSyn	17	29	26	46	59	51	71
Mild αSyn	29	19	13	15	24	15	17
Moderate αSyn	21	24	21	17	9	14	11
Severe αSyn	23	18	18	17	9	16	4
Very severe αSyn	17	17	29	12	6	9	3

Supplementary Table 7 Results of linear regressions testing the effect of confounders (independent variables applied in separate models for each confounder) on the relations between two patterns of αSyn pathology (independent variables in each model) and CA1 degeneration (dependent variable)

	Confounders						
	**Model I**: AGD Coef [95% CI]	**Model II**: ARTAG Coef [95% CI]	**Model III**: SVD temporal Coef [95% CI]	**Model IV**: Infarcts Coef [95% CI]	**Model V**: Seizure disorder Coef [95% CI]	**Model VI**: PMI Coef [95% CI]	**Model VII**: Hemisphere (left) Coef [95% CI]
(Intercept)	201.5 [145.3-257.7]	148.2 [91-205.4]	181.4 [126.8-235.9]	199.3 [143.8-254.9]	208.9 [155.3-262.6]	180.6 [119.7-241.6]	199.7 [144.8-254.6]
αSyn AmyP	**-71.1 [-95.5--46.8]*****	**-41.5 [-63.4--19.6]*****	**-48.5 [-69.3--27.7]*****	**-67.3 [-90.8--43.9]*****	**-67.9 [-88.9--47]*****	**-60.8 [-83.2--38.5]*****	**-64.8 [-86--43.7]*****
αSyn CR	-8.5 [-24.5-7.5]	9.3 [-7.6-26.1]	0.2 [-15.9-16.4]	-9.9 [-26-6.2]	-8.3 [-23.8-7.3]	-7.7 [-24-8.6]	-7.2 [-22.9-8.6]
Age at death	-0.5 [-1.2-0.3]	-0.2 [-0.9-0.5]	-0.2 [-0.9-0.5]	-0.4 [-1.2-0.3]	-0.6 [-1.3-0.1]	-0.3 [-1.1-0.4]	-0.5 [-1.2-0.2]
Sex (male)	7.2 [-6.9-21.4]	**15.4 [0.1-30.7]***	10.1 [-4.1-24.3]	5.7 [-8.4-19.7]	9.1 [-4.4-22.7]	7.5 [-6.9-21.9]	8.6 [-5.1-22.4]
Confounder	-1.5 [-23.6-20.7]	-7.5 [-26.4-11.5]	**-13.4 [-21.5--5.4]****	-2.4 [-17-12.2]	**-43.9 [-73.3--14.5]****	**0.3 [0.1-0.6]***	-12.3 [-30.8-6.2]
Sample size (n)	265	154	236	269	286	263	286
R^2	0.132	0.157	0.173	0.124	0.165	0.157	0.145
R^2 adjusted	0.115	0.128	0.155	0.108	0.15	0.141	0.129

P-values have not been corrected for multiple testing. *<0.05, **<0.01, ***<0.001

αSyn AmyP – amygdala-predominant αSyn pathology variant. αSyn CR – caudo-rostral αSyn pathology variant. PMI – postmortem interval, hemisphere – hemisphere from which tissue has been taken. SVD – small vessels disease.

Supplementary Table 8: P-values from Spearman's semi-partial correlation analysis, adjusted using the Benjamini-Hochberg method, with age at death as a covariate for αSyn-positive cases (n = 107)

	**Neuronal density CA1**	**αSyn Medulla**	**αSyn Midbrain**	**αSyn Amy**	**αSyn Temp**	**αSyn CA1**	**αSyn CA2**	**αSyn Frontal**	**αSyn GBS**	**αSyn AmyP**
Neuronal density CA1	1	0.021	0.825	0.003	0.028	0.125	0.371	0.160	0.378	1.59E-06
Medulla	0.021	1	4.69E-14	0.001	7.55E-05	1.59E-06	3.44E-09	4.84E-08	4.03E-15	1.45E-06
Midbrain	0.825	4.69E-14	1	1.65E-16	1.41E-15	9.22E-15	1.53E-16	2.19E-15	1.18E-38	0.547
Amygdala	0.003	0.001	1.65E-16	1	1.65E-30	1.05E-19	1.21E-16	4.64E-12	5.77E-31	2.01E-06
Temporal	0.028	7.55E-05	1.41E-15	1.65E-30	1	4.88E-31	1.12E-26	7.38E-15	1.44E-32	0.000
CA1	0.125	1.59E-06	9.22E-15	1.05E-19	4.88E-31	1	1.44E-32	1.41E-15	1.21E-25	0.018
CA2	0.371	3.44E-09	1.53E-16	1.21E-16	1.12E-26	1.44E-32	1	1.79E-12	1.21E-25	0.107
Frontal	0.160	4.84E-08	2.19E-15	4.64E-12	7.38E-15	1.41E-15	1.79E-12	1	3.28E-22	0.944
αSyn GBS	0.378	4.03E-15	1.18E-38	5.77E-31	1.44E-32	1.21E-25	1.21E-25	3.28E-22	1	0.349
αSyn AmyP	1.59E-06	1.45E-06	0.547	2.01E-06	0.000	0.018	0.107	0.944	0.349	1

AmyP – Amygdala-predominant αSyn pathology variant. GBS – global burden score.

Supplementary Table 9 Key differences between symptomatic Alzheimer's disease patients without αSyn and with different spreading patterns of αSyn pathology

	**no αSyn (*n* = 48)**	**Amygdala-predominant (*n* = 29)**	**Caudo-rostral (*n* = 22)**	**Observations per variable**
Age at death	80.67 (9.62)	78.21 (9.84)	80.14 (7.09)	99
CDR	2.46 (0.81)	2.83 (0.58)	2.72 (0.67)	77
αSyn GBS	0 (0)	8.21 (4.31)	9 (6.57)	99
Neuronal density CA1	126.55 (55.55)	94.51 (52.78)	129.9 (50.7)	99
Aβ Phases MTL	3.77 (0.56)	3.96 (0.19)	3.68 (0.57)	98
CERAD	2.56 (0.65)	2.38 (0.73)	2.14 (0.64)	99
Braak NFT Stages	5.25 (0.89)	5.24 (0.99)	4.86 (1.04)	99
pTau CA1	0.51 (0.23)	0.6 (0.24)	0.39 (0.21)	95
LATE-NC	0.72 (0.95)	1.34 (1.01)	1.05 (1.13)	98
CAA Severity	1.73 (0.57)	1.79 (0.49)	1.59 (0.67)	99
CAA Type 1	65%	69%	32%	99
pTDP DG	11%	38%	23%	97
Sex (Male)	42%	52%	64%	99

Variables considered as continuous have been summarized using the mean value and standard deviation (SD), while for nominal variables, we presented the percentage of cases with a positive outcome.

CAA – cerebral amyloid angiopathy. CDR – clinical dementia rating score. CERAD – consortium to establish a registry of Alzheimer's disease. DG – dentate gyrus. GBS – Global burden score. LATE-NC - limbic-predominant age-related TDP-43 encephalopathy neuropathologic change. MTL – medial temporal lobe. NFT – neurofibrillary tangle.

Supplementary table 10 Results of ANOVA and Kruskal-Wallis tests on Alzheimer's disease patients

Test	Variable	Summary
ANOVA	pTau CA1	F(2,92)=5.27, P-value=0.0068
ANOVA	Neuronal density CA1	F(2,96)=3.92, P-value=0.0231
ANOVA	Age at death	F(2,96)=0.66, P-value=0.5178
kruskal.test	αSyn global burden score	H(2)=82.99, P-value=9.52e-19
kruskal.test	LATE-NC	H(2)=6.95, P-value=0.0310
kruskal.test	CERAD	H(2)=7.14, P-value=0.0282
kruskal.test	Aβ Phases MTL	H(2)=5.33, P-value=0.0697
kruskal.test	Braak NFT Stages	H(2)=2.63, P-value=0.2688
kruskal.test	CAA Severity	H(2)=2.1, P-value=0.3505

Statistical test was applied to detect differences in parameters among Alzheimer's disease patients without αSyn pathology and with two αSyn pathology spreading patterns.

CAA – cerebral amyloid angiopathy. CERAD – consortium to establish a registry of Alzheimer's disease. LATE-NC - limbic-predominant age-related TDP-43 encephalopathy neuropathologic change. MTL – medial temporal lobe. NFT – neurofibrillary tangle.

Supplementary Table 11 Results of a post-hoc Dunn's multiple comparisons tests for Alzheimer's disease patients

Variable	Comparison	*Z*	*P.unadj*	*P.adj*	
Age at death	AmyP x αSyn-	-1.05	0.292	0.875	
Age at death	AmyP x CR	-0.48	0.633	0.949	
Age at death	αSyn- x CR	0.44	0.662	0.662	
αSyn GBS	AmyP x αSyn-	7.71	1.289e-14	3.87e-14	***
αSyn GBS	AmyP x CR	-1.6	0.875	0.875	
αSyn GBS	αSyn- x CR	-7.21	5.49e-13	8.24e-13	***
Aβ Phases MTL	AmyP x αSyn-	1.57	0.117	0.176	
Aβ Phases MTL	AmyP x CR	2.27	0.023	0.070	.
Aβ Phases MTL	αSyn- x CR	1.06	0.289	0.289	
Braak NFT Stages	AmyP x αSyn-	0.09	0.931	0.931	
Braak NFT Stages	AmyP x CR	1.43	0.153	0.230	
Braak NFT Stages	αSyn- x CR	1.49	0.136	0.409	
CAA Severity	AmyP x αSyn-	0.71	0.480	0.480	
CAA Severity	AmyP x CR	1.45	0.148	0.444	
CAA Severity	αSyn- x CR	0.94	0.345	0.518	
CERAD	AmyP x αSyn-	-1.14	0.252	0.252	
CERAD	AmyP x CR	1.47	0.141	0.212	
CERAD	αSyn- x CR	2.63	0.008	0.023	*
LATE-NC	AmyP x αSyn-	1.17	0.009	0.026	*
LATE-NC	AmyP x CR	-1.12	0.241	0.361	
LATE-NC	αSyn- x CR	-2.50	0.264	0.264	
Neuronal density CA1	AmyP x αSyn-	-2.19	0.013	0.038	*
Neuronal density CA1	AmyP x CR	-0.12	0.029	0.043	*
Neuronal density CA1	αSyn- x CR	1.35	0.904	0.904	
pTau CA1	AmyP x αSyn-	3.16	0.178	0.178	
pTau CA1	AmyP x CR	2.24	0.002	0.005	**
pTau CA1	αSyn- x CR	2.63	0.025	0.038	*

Tests were performed for variables that reached significance with ANOVA or Kruskal Wallis tests; P-values have been adjusted with the Benjamini–Hochberg method per variable; . <0.1, *<0.05, **<0.01, ***<0.001

AmyP – amygdala-predominant αSyn pathology. αSyn- - no αSyn pathology. CAA – cerebral amyloid angiopathy. CERAD – consortium to establish a registry of Alzheimer's disease. CR – caudo-rostral αSyn pathology. LATE-NC - limbic-predominant age-related TDP-43 encephalopathy neuropathologic change. MTL – medial temporal lobe. NFT – neurofibrillary tangle.

Supplementary table 12 Results of pairwise-comparisons with Fisher's exact test on nominal variables on Alzheimer's disease patients

Variable	Comparision	*P.unadj*	*P.adj*	
CAA Type I	AmyP x CR	0.012	0.028	*
CAA Type I	αSyn- x AmyP	0.805	0.805	
CAA Type I	αSyn- x CR	0.019	0.028	*
pTDP DG	AmyP x CR	0.362	0.362	
pTDP DG	αSyn- x AmyP	0.009	0.026	*
pTDP DG	αSyn- x CR	0.316	0.362	
Sex (Male)	AmyP x CR	0.569	0.569	
Sex (Male)	αSyn- x AmyP	0.480	0.569	
Sex (Male)	αSyn- x CR	0.123	0.369	

P-values have been adjusted with the Benjamini–Hochberg method per variable; . <0.1, *<0.05, **<0.01, ***<0.001

AmyP – amygdala-predominant αSyn pathology. αSyn- – no αSyn pathology. CAA – cerebral amyloid angiopathy. CR – caudo-rostral αSyn pathology. DG – dentate gyrus.

Supplementary Table 13 Results and fit statistics of the path analysis model using observations from Alzheimer's disease patients (*n* = 95)

**Regressions**					
	Estimate	*Std. Estimate*	*SE*	*z*	*P*
LATE-NC ~					
αSyn AmyP	0.226	0.228	0.099	2.29	0.022*
pTau CA1 ~					
αSyn AmyP	0.244	0.247	0.098	2.49	0.013*
Neuronal Density CA1 ~					
pTau CA1	-0.382	-0.373	0.09	-4.25	<0.001
LATE-NC	-0.35	-0.343	0.089	-3.92	<0.001
αSyn AmyP	-0.097	-0.096	0.091	-1.06	0.287
**Variances**					
	Estimate	*Std. Estimate*	*SE*	*z*	*P*
LATE-NC	0.929	0.939	0.135	6.89	< 0.001
pTau CA1	0.94	0.948	0.136	6.89	< 0.001
Neuronal Density CA1	0.712	0.687	0.103	6.89	< 0.001
**Fit statistics**					
Measure	Obtained values	Good fit criterium[2]			
Test statistic	0.304				
Degrees of freedom	1				
P (Chi-square)	0.581	>0.05			
Comparative Fit Index (CFI)	1	>0.95			
Tucker-Lewis Index (TLI)	1.1	>0.95			
RMSEA [90% CI]	0 [0, 0.22]	<0.08			
SRMR	0.02	<0.05			

SE – standard error, Std – standardized values

AmyP – amygdala-predominant αSyn pathology. LATE-NC - limbic-predominant age-related TDP-43 encephalopathy neuropathologic change. RMSEA – root mean square error of approximation. SRMR – standardized root mean square residual

Supplementary references

1. McKeith IG, Dickson DW, Lowe J, et al. Diagnosis and management of dementia with Lewy bodies: third report of the DLB Consortium. *Neurology*. 2005;65(12):1863-1872. doi:10.1212/01.wnl.0000187889.17253.b1

2. Montine TJ, Phelps CH, Beach TG, et al. National Institute on Aging-Alzheimer's Association guidelines for the neuropathologic assessment of Alzheimer's disease: a practical approach. *Acta Neuropathol (Berl)*. 2012;123(1):1-11. doi:10.1007/s00401-011-0910-3

3. Hooper D, Coughlan J, Mullen M. Structural Equation Modeling: Guidelines for Determining Model Fit. *Electron J Bus Res Methods*. 2007;6.

R Code

Custom functions

```r
library(dplyr)
library(ggplot2)
library(data.table)
library(FSA)
library(rstatix)

## Function that rounds p-values to three decimal places or
## changes them to scientific notation if they are smaller
## than parameter round_no
round_pval <- function(pval, pval_threshold = 0.001, round_no = 4) {

    pval_rounded <- paste0(ifelse(pval < pval_threshold, format(pval,
        scientific = TRUE, digits = round_no), format(round(pval,
        round_no), nsmall = round_no)))
    return(pval_rounded)

}

## Function that checks if a column is binary
is_binary <- function(data, col_name) {

    unique_values <- unique(data[[col_name]])
    unique_values <- unique_values[!is.na(unique_values)]
    return(all(sort(unique_values) == c(0, 1)) && length(unique_values) ==
        2)

}

## FUNCTION to perform normality check and then ANOVA or
## Kruskal-Wallis tests Dependent - continuous/ordinal
## variable (cont_vars) Independent - categorical variable
## with three or more groups (group_var)

perform_anova_KW <- function(data, cont_vars, group_var) {

    # Prepare empty dataframes
    results_main <- data.table(Test = character(0), variable = character(0),
        Summary = character(0), n = numeric(0), significant = character(0),
        P_value = numeric(0), P_value_rounded = numeric(0), F_df = character(0),
        F_value = numeric(0), H = numeric(0), df = numeric(0),
        Order = numeric(0))

    results_shapiro <- data.table()
```

```
results_posthoc <- data.table(Comparison = character(0),
    Z = numeric(0), P.unadj = numeric(0), P.adj = numeric(0),
    variable = character(0), summary = character(0))

# Loop through each variable
count = 0
for (cont_var in cont_vars) {

    subset_data <- na.omit(data[, c(cont_var, group_var)])

    shapiro_p_value <- 0
    count = count + 1

    tryCatch({
        # Check for normality using the Shapiro-Wilk
        # test Try catch because for some groups the
        # values are the same and the test won't work
        shapiro_test <- subset_data %>%
            group_by(!!sym(group_var)) %>%
            summarise(shapiro_p_value = shapiro.test(!!sym(cont_var))$p.value) %>%
            mutate(variable = cont_var)

        shapiro_p_value <- shapiro_test$shapiro_p_value

    }, error = function(e) {
        # If error then variable is not normal keep
        # shapiro_p_value as 0 and store info that it
        # was an error
        cat(paste0(cont_var, " :error: Identical values in the dataset.\n"))
        matrix_empty = matrix(nrow = length(unique(subset_data[,
            group_var])), ncol = 3)
    })

    # Add result of normality test to the data table
    results_shapiro <- data.table::rbindlist(list(shapiro_test,
        as.data.table(results_shapiro)), use.names = FALSE)

    # Perform ANOVA only if the data are normally
    # distributed (p-value > 0.05)
    if (all(shapiro_p_value >= 0.05)) {
        # Perform ANOVA test
        anova_result <- anova(lm(formula(paste(cont_var,
            "~", group_var)), data = subset_data))

        # Extract relevant information from the ANOVA
        # result
        df1 <- anova_result$Df[1]
        df2 <- anova_result$Df[2]
        f_value <- round(anova_result$F[1], 2)
        P_value <- anova_result$Pr[1]
        P_value_rounded <- round_pval(P_value)
        n <- nrow(na.omit(subset_data))
        summary_str <- paste0("F(", df1, ",", df2, ")=",
```

```
            round(f_value, 2), ", P-value=", P_value_rounded)

        # Add results to the result_table
        results_main <- rbind(results_main, data.table(F_df = paste(df1,
            df2, sep = ", "), F_value = f_value, P_value = P_value,
            P_value_rounded = P_value_rounded, Summary = summary_str,
            n = n, variable = cont_var, Test = "ANOVA", Order = count),
            fill = TRUE)
    } else {

        # Perform Kruskal-Wallis test if variable is
        # non-normally distributed
        kw_results <- kruskal.test(formula(paste(cont_var,
            "~", group_var)), data = subset_data)

        # Extract relevant information Kruskal-Wallis
        # test
        H <- round(kw_results$statistic, 2)
        df <- kw_results$parameter
        P_value <- kw_results$p.value
        P_value_rounded <- round_pval(P_value)
        n <- nrow(na.omit(subset_data))
        summary_str <- paste0("H(", df, ")=", H, ", P-value=",
            P_value_rounded)

        results_main <- rbind(results_main, data.table(H = H,
            df = df, P_value = P_value, P_value_rounded = P_value_rounded,
            Summary = summary_str, n = n, variable = cont_var,
            Test = "kruskal.test", Order = count), fill = TRUE)
    }

    # Perform post-hoc test
    subset_data[[group_var]] <- as.factor(subset_data[[group_var]])
    posthoc_DT = dunnTest(formula(paste(cont_var, "~", group_var)),
        data = subset_data, method = "bh")

    # Add results of post-hoc test to the post-hoc
    # dataframe
    posthoc_DT_results <- posthoc_DT$res
    posthoc_DT_results$Z <- round(posthoc_DT_results$Z, 2)
    posthoc_DT_results$variable <- cont_var
    posthoc_DT_results$summary <- paste0("Z=", round(posthoc_DT_results$Z,
        2), ", P-value=", round_pval(posthoc_DT_results$P.adj))

    results_posthoc <- data.table::rbindlist(list(posthoc_DT_results,
        as.data.table(results_posthoc)), use.names = FALSE)
}

# Indicate significant associations
results_posthoc$significant <- ifelse(results_posthoc[, c("P.adj")] <
    0.05, "YES", "NO")
results_main$significant <- ifelse(results_main[, c("P_value")] <
    0.05, "YES", "NO")
```

```r
    # Sort from the smallest to the largest p-values
    results_posthoc <- results_posthoc[order(P.adj), ]
    results_main <- results_main[order(P_value), ]

    # Round p-values and z score for publication
    results_shapiro[, 2] <- round_pval(results_shapiro[, 2])
    results_posthoc[, c("P.unadj")] <- round_pval(results_posthoc[,
        c("P.unadj")])
    results_posthoc[, c("P.adj")] <- round_pval(results_posthoc[,
        c("P.adj")])
    results_posthoc[, c("Z")] <- round_pval(results_posthoc[,
        c("Z")])

    # Put all results into one list
    results <- list(results_main = results_main, results_posthoc = results_posthoc,
        results_shapiro = results_shapiro)

    return(results)
}

## FUNCTION that take groups data based on one variable and
## creates summary statistics for each of group for
## continuous and binary variables
group_summary <- function(data, group_var, cont_vars, binary_vars,
    round_no = 2) {
    # Group the dataframe by the variables in group_var
    grouped_data <- aggregate(data[, cont_vars], data[, group_var,
        drop = FALSE], FUN = function(x) paste0(round(mean(x,
        na.rm = TRUE), round_no), " (", round(sd(x, na.rm = TRUE),
        round_no), ")"))

    # Calculate percentage of ones for categorical columns
    cat_summary <- aggregate(data[, binary_vars], data[, group_var,
        drop = FALSE], FUN = function(x) round(mean(x == 1, na.rm = TRUE),
        round_no))

    # Count the number of observations per group
    obs_counts <- aggregate(data[, 1], data[, group_var, drop = FALSE],
        FUN = length)
    colnames(obs_counts) <- c(group_var, "n")

    # Merge the summary results
    summary_data <- merge(grouped_data, cat_summary, by = group_var)
    summary_data <- merge(summary_data, obs_counts, by = group_var)

    # Rename the binary_vars columns to indicate
    # percentages binary_colnames <- paste0(binary_vars,
    # '_percent') colnames(summary_data)[match(binary_vars,
    # colnames(summary_data))] <- binary_colnames

    return(summary_data)
}
```

```r
## FUNCTION that takes the model generated by linear
## regression as an input and creates nice table with all
## information
create_lm_summary_table <- function(lm_object, robust_coef = FALSE,
    round_stat = 1, round_pval = 3, round_R2 = 3) {

    # If specified, replace coefficients with robust
    # coefficients
    if (length(robust_coef) > 1) {
        lm_object_coef <- robust_coef

    } else {
        lm_object_coef <- summary(lm_object)$coefficients
    }

    # Extract coefficients, their standard errors and
    # t-values
    coef_data <- data.frame(Coef = round(lm_object_coef[, "Estimate"],
        round_stat), SE = round(lm_object_coef[, "Std. Error"],
        round_stat), t_value = round(lm_object_coef[, "t value"],
        round_stat))

    # Calculate 95% confidence intervals
    ci_data <- cbind(lower = round(confint(lm_object)[, 1], round_stat),
        upper = round(confint(lm_object)[, 2], round_stat))
    coef_data$CI_95 <- apply(ci_data, 1, function(x) paste0("[",
        x[1], "-", x[2], "]"))

    # Extract p-values for coefficients
    p_value_coef <- lm_object_coef[, "Pr(>|t|)"]
    P_value_coef_rounded <- paste0(ifelse(p_value_coef < 0.001,
        format(p_value_coef, scientific = TRUE, digits = 3),
        format(round(p_value_coef, round_pval), nsmall = 3)))
    coef_data$P_value_coef <- P_value_coef_rounded

    # Create the coef_summary column
    coef_data$coef_summary <- apply(coef_data, 1, function(x) paste0(" =",
        x[1], ", P-value=", x[5]))
    coef_data$coef_summary_short <- apply(coef_data, 1, function(x) paste(x[1],
        x[4]))

    # Extract model summary information
    model_summary <- summary(lm_object)
    model_f <- summary(lm_object)$fstatistic
    p_value_model <- pf(model_f[1], model_f[2], model_f[3], lower.tail = F)
    p_value_model_rounded <- paste0(ifelse(p_value_model < 0.001,
        format(p_value_model, scientific = TRUE, digits = 3),
        format(round(p_value_model, round_pval), nsmall = 3)))

    model_data <- data.frame(R2.adj = round(model_summary$adj.r.squared,
        round_R2), R2 = round(model_summary$r.squared, round_R2),
        P_value_model = p_value_model_rounded, F = round(model_summary$fstatistic[1],
            round_stat), df = paste0(model_summary$fstatistic[2],
```

```r
            ", ", model_summary$fstatistic[3]))

    # Create the model_summary column
    model_data$model_summary <- paste0("F(", model_data$df[1],
        ") =", model_data$F, ", P-value=", model_data$P_value_model)
    model_data <- model_data[rep(1, nrow(coef_data)), ]

    # Combine all information into the final table
    result_table <- cbind(coef_data, model_data)
    result_table <- result_table[, c("Coef", "CI_95", "SE", "t_value",
        "P_value_coef", "coef_summary", "coef_summary_short",
        "R2", "R2.adj", "P_value_model", "F", "df", "model_summary")]
    data.table::setDT(result_table, keep.rownames = TRUE)[]
    return(result_table)
}

## FUNCTION that perform Fisher's test for binary data
perform_fishers_test <- function(data, binary_vars, group_var) {

    # Prepare empty dataframes
    results_main <- data.frame()
    results_posthoc <- data.frame()
    results_contingency_tables <- data.frame()

    # Loop through all binary variables
    count <- 0
    for (binary_var in binary_vars) {
        # Create a contingency table
        contingency_table <- table(data[[binary_var]], data[[group_var]])
        count = count + 1

        # Perform Fisher's test to see if there is a
        # difference between any group
        fisher_test_result <- rstatix::fisher_test(contingency_table,
            detailed = FALSE)
        fisher_test_result$p_value_rounded <- round_pval(fisher_test_result$p)
        fisher_test_result$variable <- binary_var
        fisher_test_result$Order <- count

        # Perform Fisher's pairwise tests to see
        # differences between each of the group combination
        fisher_pairwise_test_result <- rstatix::pairwise_fisher_test(contingency_table,
            p.adjust.method = "BH", detailed = FALSE)

        fisher_pairwise_test_result$p_value_rounded <- round_pval(fisher_pairwise_test_result$p.adj)
        fisher_pairwise_test_result$variable <- binary_var
        contingency_table <- as.data.frame(contingency_table)
        contingency_table$variable <- binary_var

        fisher_pairwise_test_result <- fisher_pairwise_test_result[,
            c(8, seq(1:7))]

        # Add obtained results to dataframes storing
```

```
        # informaiton for all variables
        results_main <- data.table::rbindlist(list(fisher_test_result,
            as.data.table(results_main)), use.names = FALSE)

        results_posthoc <- data.table::rbindlist(list(fisher_pairwise_test_result,
            as.data.table(results_posthoc)), use.names = FALSE)

        results_contingency_tables <- data.table::rbindlist(list(contingency_table,
            as.data.table(results_contingency_tables)), use.names = FALSE)
    }
    # Sort results based on p-value
    results_main <- results_main[order(p), ]
    results_posthoc <- results_posthoc[order(p.adj), ]

    # Put all results into one list
    results <- list(results_main = results_main, results_posthoc = results_posthoc,
        results_contingency_tables = results_contingency_tables)

    return(results)

}
```

Data preparation

```
# EXCLUSION CRITERIA Exclude cases with any FTLD
# neuropathology
cases <- cases[cases$FTLD_neuropathology == 0, ]

# Exclude cases with ALS
cases <- cases[cases$Klinik_ALS == 0, ]

# Exclude cases with Multiple System Atrophy
cases <- cases[is.na(cases$MSA_YN), ]

# Exclude cases younger than 50 years old
minimum_age <- 50
cases <- cases[!cases$AGE < minimum_age, ]

# Exclude cases with other severe neurological conditions
# or lack of tissue
cases <- cases[cases$EXCLUSION_other_and_tissue == "NO", ]

# Exclude cases without dementia diagnosis
cases <- cases[!is.na(cases$DEMENTIA), ]

# Remove cases without information about aSyn or ADNC
cases <- cases[!is.na(cases$Braak_PD), ]
cases <- cases[!is.na(cases$NIA_AA), ]

# Use the same cases (for revisions)
cases <- cases[cases$aSyn_article == "YES", ]
```

```r
# Convert columns to numeric
cases$Braak_PD <- as.numeric(cases$Braak_PD)
cases$AGE <- as.numeric(cases$AGE)
cases$PMI <- as.numeric(cases$PMI)

# Create new variables and clean existing ones for further
# analysis
cases <- cases %>%
    mutate(AD_YN = ifelse(NIA_AA >= 2, 1, 0)) %>%
    mutate(aSyn_YN = ifelse(Braak_PD > 0, 1, 0)) %>%
    mutate(sex_binary = ifelse(SEX == "m", 1, 0)) %>%
    mutate(CAA1_YN = ifelse(CAA_TYPE == 1, 1, 0)) %>%
    mutate(AGD = ifelse(AGD == 2, 1, AGD)) %>%
    mutate(pTau_positive_neurons_CA1_ST_KG = ifelse(!is.na(cases$pTau_positive_neurons_CA1_KG),
        cases$pTau_positive_neurons_CA1_KG, cases$pTau_positive_neurons_CA1_ST))

# ASYN INTERPOLATION

# Define regions with aSyn pathology
asyn_regions <- c("asyn_med", "asyn_mibr", "asyn_amy", "asyn_temp_post",
    "asyn_CA2", "asyn_CA1", "asyn_front")

# Set the allowed number of missing values for
# interpolation
missing_val_allowed <- 1

# Extract columns for interpolation
cases_interpolate <- cases[, c(asyn_regions)]

# Identify cases with enough data for interpolation
row_exceeds_threshold <- rowSums(is.na(cases_interpolate)) <=
    missing_val_allowed

# Interpolate missing values
cases_interpolated <- zoo::zoo(t(cases_interpolate[row_exceeds_threshold,
    ]))
cases_interpolated <- t(round(zoo::na.approx(cases_interpolated)))

# Count interpolated values
no_missing_before <- rowSums(is.na(cases_interpolate)) == 0
no_missing_after <- rowSums(is.na(cases_interpolated)) == 0
interpolated_no <- sum(no_missing_after) - sum(no_missing_before)
interpolated_perc <- interpolated_no/(nrow(cases_interpolated) *
    length(asyn_regions))

# Replace missing values with interpolated ones
cases[row_exceeds_threshold, asyn_regions] <- cases_interpolated

# ASYN PATTERNS Calculate aSyn severity ratio and divide
# cases into two aSyn patterns
cases <- cases %>%
    mutate(asyn_mtl_bst_ratio = (asyn_amy + asyn_temp_post)/(asyn_med +
        asyn_mibr)) %>%
```

```
    mutate(asyn_pattern = ifelse(aSyn_YN == 0, "aSyn-", ifelse(asyn_mtl_bst_ratio >
        1, "Amygdala-predominant", ifelse(asyn_mtl_bst_ratio <=
        1, "Caudo-rostral", 0)))) %>%
    mutate(asyn_pattern_cr = ifelse(asyn_pattern == "Caudo-rostral",
        1, 0)) %>%
    mutate(asyn_pattern_amy = ifelse(asyn_pattern == "Amygdala-predominant",
        1, 0))

# Convert asyn_pattern to a factor, set order of levels
cases$asyn_pattern <- factor(cases$asyn_pattern, levels = c("aSyn-",
    "Amygdala-predominant", "Caudo-rostral"))

# Calculate global severity score for aSyn
cases$asyn_GSS <- rowSums(cases[, c("asyn_med", "asyn_mibr",
    "asyn_amy", "asyn_temp_post", "asyn_front")], na.rm = FALSE)
cases$asyn_GSS[cases$Braak_PD == 0] <- 0

# How many cases was excluded?
print(paste0("Number of aSyn positive cases = ", nrow(cases[cases$aSyn_YN ==
    1, ])))
print(paste0("Number of excluded aSyn positive cases = ", nrow(cases[cases$aSyn_YN ==
    1, ]) - nrow(cases[cases$aSyn_YN == 1 & !is.na(cases$asyn_pattern),
    ])))

# Remove cases without aSyn pattern
cases <- cases[!is.na(cases$asyn_pattern), ]

# DEFINE GROUPS Define group of cases with aSyn positivity
# and no missing aSyn severity scores
cases_aSyn <- cases[cases$aSyn_YN == 1, ]

# Define group of symptomatic AD cases with known aSyn
# pattern
cases$high_evidence_dementia[is.na(cases$high_evidence_dementia)] <- 0
cases_AD <- cases[cases$AD_YN == 1 & cases$high_evidence_dementia ==
    1, ]
```

Interrater agreement

```
counts_both_raters <- cases[cases$CA1_density_rater == "KG_SO",
    c("Neuronal_Density_CA1_mm2_pTDP_KG", "Neuronal_Density_CA1_mm2_pTDP_ST")]

# Calculate Pearson correlation
corx::corx(as.matrix(counts_both_raters), method = "pearson",
    )

# Calculate Intraclass Correlation Coefficient (ICC)
irr::icc(counts_both_raters, model = "twoway", type = "agreement",
    unit = "single")
```

Supplementary Table 1

```r
# Descriptive statistics TABLE all cases
grouping_var <- c("aSyn_YN", "NIA_AA")

cont_vars <- c("AGE", "CDR", "Braak_NFT", "ABMTL_OC", "CERAD",
    "Neuronal_Density_CA1_mm2_pTDP_KG", "Braak_PD", "asyn_GSS",
    "PMI", "svd_temporal_semiq")

binary_vars <- c("asyn_pattern_amy", "sex_binary", "siezure_disorder_clinic",
    "high_evidence_dementia", "AGD", "ARTAG", "INFARCTI", "left_hemisphere_YN",
    "pTDP43_AH_post_YN")

titles <- c("Age at death", "CDR", "Braak NFT Stages", "A  Phases MTL",
    "CERAD", "Neuronal density CA1", "Braak PD Stages", "aSyn GBS",
    "PMI", "Arteriolosclerosis temporal", "aSyn AmyP", "Sex (Male)",
    "Epilepsy", "Dementia", "AGD", "ARTAG", "Infarcts", "Hemisphere (left)",
    "pTDP43_AH_post_YN", "n")

# Generate summary statistics
summary_table_all <- group_summary(cases, grouping_var, cont_vars,
    binary_vars, round_no = 2)
summary_table_all <- as.data.frame(summary_table_all)
colnames(summary_table_all) <- c(grouping_var[1], grouping_var[2],
    titles)
summary_table_all <- as.data.frame(t(summary_table_all))

# Save summary statistics to Excel file
openxlsx::write.xlsx(summary_table_all, file = paste0(save_path,
    "ST1_summary_info_all_cases.xlsx"), rowNames = TRUE)
```

Figure 1

```r
library(readr)
library(gridExtra)

sufix <- "boxplot"

var_names <- c("Neuronal_Density_CA1_mm2_pTDP_KG")

title <- "Neuronal density CA1 (mm2)"

lim <- 350

base_size <- 70
line_width <- base_size/30
summary_size <- base_size/2
boxplot_width <- 0.35
bar_width <- 0.35
line_color <- "grey20"
strip_text_size <- 0.9
```

```
lb_colors <- c("steelblue4", "darkorange3", "orange")
ratio_col <- colorRampPalette(c("orange", "darkorange3"))

labels_ADNC <- c(`0` = "No ADNC", `1` = "Mild ADNC", `2` = "Moderate ADNC",
    `3` = "High ADNC")

cases$asyn_pattern <- as.factor(cases$asyn_pattern)
# BOXPLOT of MTL/brainstem ratio depending on ADNC
F1_A <- ggplot(cases_aSyn, aes(x = as.factor(NIA_AA), y = asyn_mtl_bst_ratio,
    fill = as.factor(NIA_AA))) + geom_boxplot(size = line_width,
    width = boxplot_width, color = line_color, position = position_dodge(width = 0.005)) +
    geom_point(position = position_jitter(seed = 4, width = 0.1),
        size = 12, alpha = 1, color = "#595959") + theme_minimal(base_size = base_size) +
    theme(axis.title.x = element_blank(), axis.ticks.x = element_blank(),
        legend.position = "none", axis.text.x = element_text(size = base_size *
            strip_text_size)) + scale_fill_manual(values = ratio_col(4)) +
    ylab(" Syn limbic/brainstem ratio") + scale_x_discrete(labels = labels_ADNC) +
    geom_hline(yintercept = 1, linetype = 2, col = "black", linewidth = 2)

# BOXPLOT of aSyn GSS depending on ADNC
F1_B <- ggplot(cases_aSyn, aes(x = as.factor(NIA_AA), y = asyn_GSS,
    fill = as.factor(NIA_AA))) + geom_boxplot(size = line_width,
    width = boxplot_width, color = line_color, position = position_dodge(width = 0.005)) +
    geom_point(position = position_jitter(seed = 3, width = 0.1),
        size = 10, alpha = 1, color = "#595959") + theme_minimal(base_size = base_size) +
    theme(axis.title.x = element_blank(), axis.ticks.x = element_blank(),
        legend.position = "none", axis.text.x = element_text(size = base_size *
            strip_text_size)) + scale_fill_manual(values = ratio_col(4)) +
    ylab(" Syn global burden score") + scale_x_discrete(labels = labels_ADNC) +
    ylim(0, 30)



dist_colors <- c("steelblue4", "darkorange3", "orange")

F1_C <- ggplot(cases[!is.na(cases$asyn_pattern), ], aes(x = Neuronal_Density_CA1_mm2_pTDP_KG,
    fill = asyn_pattern, color = asyn_pattern)) + geom_density(color = "black",
    alpha = 0.3, size = 1) + theme_minimal(base_size = 120) +
    scale_fill_manual(values = dist_colors) + xlab("Neuronal density CA1 (mm2)") +
    theme(plot.title = element_blank(), legend.position = "none")
```

Save plot for Figure 1

```
# Save Figure 1A-B
tiff(paste0(save_path, "Figure1AB_raw.tiff"), units = "in", width = 60,
    height = 20, res = 300, compression = "lzw")
pp2 <- list(F1_A, F1_B)
margin = theme(plot.margin = unit(c(t = 4, r = 8, b = 0, l = 2),
    "cm"))
do.call(grid.arrange, c(lapply(pp2, "+", margin), ncol = 2))
dev.off()
```

```
# Save Figure 1C
tiff(paste0(save_path, "Figure1C2_raw.tiff"), units = "in", width = 30,
    height = 20, res = 300, compression = "lzw")
F1_C
dev.off()
```

Statistics Figure 1A-B

```
# replace the infinity values from ratio with a very large
# number
cases_aSyn$asyn_mtl_bst_ratio[cases_aSyn$asyn_mtl_bst_ratio ==
    Inf] <- 1e+09
cases_aSyn$asyn_mtl_bst_ratio <- as.numeric(cases_aSyn$asyn_mtl_bst_ratio)

cols_names <- c("asyn_mtl_bst_ratio", "NIA_AA", "AGE")
spcorr <- corx::corx(cases_aSyn[, c(cols_names)], z = c("AGE"),
    method = "spearman")
spcorr
spcorr$p

cols_names <- c("asyn_GSS", "NIA_AA", "AGE")
spcorr <- corx::corx(cases_aSyn[, c(cols_names)], z = c("AGE"),
    method = "spearman")
spcorr
spcorr$p
```

Statistics Figure 1C

```
# Descriptive statistics neuronal density CA1 for people
# with and without aSyn
paste0(round(mean(cases[cases$asyn_pattern == "aSyn-", ]$Neuronal_Density_CA1_mm2_pTDP_KG),
    1), "(", round(sd(cases[cases$asyn_pattern == "aSyn-", ]$Neuronal_Density_CA1_mm2_pTDP_KG),
    1), ")")

paste0(round(mean(cases[cases$asyn_pattern == "Caudo-rostral",
    ]$Neuronal_Density_CA1_mm2_pTDP_KG), 1), "(", round(sd(cases[cases$asyn_pattern ==
    "Caudo-rostral", ]$Neuronal_Density_CA1_mm2_pTDP_KG), 1),
    ")")

paste0(round(mean(cases[cases$asyn_pattern == "Amygdala-predominant",
    ]$Neuronal_Density_CA1_mm2_pTDP_KG), 1), "(", round(sd(cases[cases$asyn_pattern ==
    "Amygdala-predominant", ]$Neuronal_Density_CA1_mm2_pTDP_KG),
    1), ")")


# Check normality of data in both groups
shapiro.test(as.numeric(cases$Neuronal_Density_CA1_mm2_pTDP_KG[cases$asyn_pattern ==
    "aSyn-"]))
shapiro.test(as.numeric(cases$Neuronal_Density_CA1_mm2_pTDP_KG[cases$asyn_pattern ==
    "Caudo-rostral"]))
```

```r
shapiro.test(as.numeric(cases$Neuronal_Density_CA1_mm2_pTDP_KG[cases$asyn_pattern ==
    "Amygdala-predominant"]))

# Perform Wilcoxon test
kruskal.test(formula(paste("Neuronal_Density_CA1_mm2_pTDP_KG",
    "~", "asyn_pattern")), data = cases)

dunnTest(formula(paste("Neuronal_Density_CA1_mm2_pTDP_KG", "~",
    "asyn_pattern")), data = cases, method = "bh")
```

Supplementary Table 6

```r
library(dplyr)
library(tidyr)
# Frequency of aSyn severities across regions Vector
# containing the names of factor columns
asyn_regions <- c("asyn_med", "asyn_mibr", "asyn_amy", "asyn_temp_post",
    "asyn_CA1", "asyn_CA2", "asyn_front")
cases_aSyn_all_summary <- cases[cases$ID %in% cases_aSyn, ]

# Create a summary dataframe
asyn_values_table <- sapply(X = cases_aSyn[, asyn_regions], FUN = table)

openxlsx::write.xlsx(as.data.frame(asyn_values_table), file = paste0(save_path,
    "ST6_asyn_severity_frequencies.xlsx"), showNA = FALSE)
```

##Supplementary Table 7

```r
# Linear regressions with potential confounders
# Argyrophilic grain disease
model_density_AGD <- lm(Neuronal_Density_CA1_mm2_pTDP_KG ~ asyn_pattern +
    AGE + SEX + AGD, data = cases)

# Arteriolosclerosis in temporal lobe
model_density_SVD <- lm(Neuronal_Density_CA1_mm2_pTDP_KG ~ asyn_pattern +
    AGE + SEX + svd_temporal_semiq, data = cases)

# Presence of infarction
model_density_INF <- lm(Neuronal_Density_CA1_mm2_pTDP_KG ~ asyn_pattern +
    AGE + SEX + INFARCTI, data = cases)

# Aging-related tau astrogliopathy
model_density_asyn_ARTAG <- lm(Neuronal_Density_CA1_mm2_pTDP_KG ~
    asyn_pattern + AGE + SEX + ARTAG, data = cases)

# Seizure disorder diagnosis
model_density_seizure <- lm(Neuronal_Density_CA1_mm2_pTDP_KG ~
    asyn_pattern + AGE + SEX + siezure_disorder_clinic, data = cases)

# Postmortem interval
model_density_PMI <- lm(Neuronal_Density_CA1_mm2_pTDP_KG ~ asyn_pattern +
```

```
    AGE + SEX + PMI, data = cases)

# Used hemisphere
model_density_hemisp <- lm(Neuronal_Density_CA1_mm2_pTDP_KG ~
    asyn_pattern + AGE + SEX + left_hemisphere_YN, data = cases)

# Create summary tables of results
model_density_AGD.summary <- create_lm_summary_table(model_density_AGD,
    round_stat = 1)
model_density_AGD.summary$n <- length(model_density_AGD$residuals)

model_density_SVD.summary <- create_lm_summary_table(model_density_SVD,
    round_stat = 1)
model_density_SVD.summary$n <- length(model_density_SVD$residuals)

model_density_INF.summary <- create_lm_summary_table(model_density_INF,
    round_stat = 1)
model_density_INF.summary$n <- length(model_density_INF$residuals)

model_density_asyn_ARTAG.summary <- create_lm_summary_table(model_density_asyn_ARTAG,
    round_stat = 1)
model_density_asyn_ARTAG.summary$n <- length(model_density_asyn_ARTAG$residuals)

model_density_seizure.summary <- create_lm_summary_table(model_density_seizure,
    round_stat = 1)
model_density_seizure.summary$n <- length(model_density_seizure$residuals)

model_density_PMI.summary <- create_lm_summary_table(model_density_PMI,
    round_stat = 1)
model_density_PMI.summary$n <- length(model_density_PMI$residuals)

model_density_hemisp.summary <- create_lm_summary_table(model_density_hemisp,
    round_stat = 1)
model_density_hemisp.summary$n <- length(model_density_hemisp$residuals)

results_lm_all <- list(model_density_AGD = model_density_AGD.summary,
    model_density_SVD = model_density_SVD.summary, model_density_INF = model_density_INF.summary,
    model_density_asyn_ARTAG = model_density_asyn_ARTAG.summary,
    model_density_seizure = model_density_seizure.summary, model_density_PMI = model_density_PMI.summar
    model_density_hemisp = model_density_hemisp.summary)
# Save tables
openxlsx::write.xlsx(results_lm_all, file = paste0(save_path,
    "ST7_summary_all_models.xlsx"), showNA = FALSE)
```

Table 1

```
# Multiple linear regression aSyn with spreading patterns,
# pTDP, ADNC
model_density_patterns <- lm(Neuronal_Density_CA1_mm2_pTDP_KG ~
    AGE + SEX + Braak_NFT + ABMTL_OC + asyn_pattern + pTDP43_AH_post_YN,
    data = cases)
```

```
# Get summary of the mdel
model_density_patterns.summary <- create_lm_summary_table(model_density_patterns)
model_density_patterns.summary$n <- length(model_density_patterns$residuals)

# Save the summary of the model
openxlsx::write.xlsx(model_density_patterns.summary, file = paste0(save_path,
    "T1_summary_pattern_model.xlsx"), showNA = FALSE)
```

Figure 2, Supplementary Table 8

```
# Define a color palette for the correlation plot
col <- colorRampPalette(c("darkorange2", "darkorange", "#FFFFFF",
    "lightblue3", "steelblue4"))

# Set the global size for correlation text
global_size_corr <- 6

# Define the regions and covariates to be used in the
# correlation analysis
asyn_regions <- c("asyn_med", "asyn_mibr", "asyn_amy", "asyn_temp_post",
    "asyn_CA1", "asyn_CA2", "asyn_front", "asyn_GSS", "asyn_pattern_amy")
covariates <- c("AGE")
cols_names <- c("Neuronal_Density_CA1_mm2_pTDP_KG", asyn_regions,
    covariates)

# Define the labels for the correlation plot
glabels <- c(" Neuronal \n  density CA1", "Medulla", "Midbrain",
    "Amygdala", "Temporal", "CA1", "CA2", "Frontal", " Syn GBS",
    " Syn AmyP", "Age")

# Filter the cases to only include those with aSyn
# pathology
cases_aSyn_corr <- cases_aSyn[, c(cols_names)]

# Perform correlation analyses
corr <- corx::corx(as.matrix(cases_aSyn_corr), method = "spearman",
    )
pcorr <- ppcor::pcor(na.omit(cases_aSyn_corr), method = "spearman")
spcorr <- corx::corx(cases_aSyn_corr, z = covariates, method = "spearman")

# Extract the correlation values and p-values
r_val <- spcorr$r
p_val <- spcorr$p
p_val_adj <- matrix(p.adjust(p_val, method = "BH"), nrow = nrow(p_val))

# Add nice variable names to the correlation matrix
colnames(r_val) <- head(glabels, -length(covariates))
rownames(r_val) <- head(glabels, -length(covariates))
colnames(p_val_adj) <- head(glabels, -length(covariates))
rownames(p_val_adj) <- head(glabels, -length(covariates))
```

```r
# Create the correlation plot with custom colors and
# coefficient values
tiff(paste0(save_path, "Figure2_coef_raw.tiff"), units = "in",
    width = 40, height = 40, res = 300, compression = "lzw")
corrplot::corrplot(r_val, method = "color", addCoef.col = "black",
    col = col(200), type = "lower", pch.col = "black", pch.cex = 2,
    tl.col = "black", tl.srt = 45, tl.cex = global_size_corr,
    cl.cex = global_size_corr, number.cex = global_size_corr)
dev.off()

# Create the correlation plot with significance stars
tiff(paste0(save_path, "Figure2_stars_raw.tiff"), units = "in",
    width = 40, height = 40, res = 300, compression = "lzw")
corrplot::corrplot(r_val, method = "shade", shade.col = NA, p.mat = p_val_adj,
    insig = "label_sig", sig.level = c(0.001, 0.01, 0.05), col = col(200),
    type = "lower", pch.col = "black", pch.cex = 4, tl.col = "black",
    tl.srt = 45, tl.cex = global_size_corr, cl.cex = global_size_corr)
dev.off()

# Create a matrix with the correlation coefficients and
# adjusted p-values
full_corr_matrix <- matrix(paste(" =", round(r_val, 2), ", p-value=",
    ifelse(p_val_adj < 0.001, format(p_val_adj, scientific = TRUE,
        digits = 3), format(round(p_val_adj, 3), nsmall = 3)),
    sep = ""), nrow = nrow(r_val))

colnames(full_corr_matrix) <- head(glabels, -length(covariates))
rownames(full_corr_matrix) <- head(glabels, -length(covariates))

# Prepare the results for Supplementary Table 6
results_corr_lb <- list(summary = full_corr_matrix, p_val_adj = p_val_adj,
    p_val = p_val, coef = r_val)

# Save the correlation results to an Excel file
openxlsx::write.xlsx(results_corr_lb, file = paste0(save_path,
    "ST8_summary_correlation_lb.xlsx"), showNA = FALSE)
```

Figure 3, Supplementary Table 9, 10, 11, 12

```r
library(gridExtra)

group_var <- "asyn_pattern"

# Variables to be plotted
plot_vars <- c("AGE", "sex_binary", "asyn_GSS", "Neuronal_Density_CA1_mm2_pTDP_KG",
    "ABMTL_OC", "CERAD", "Braak_NFT", "pTau_positive_neurons_CA1_ST_KG",
    "CAA1_YN", "CAA_SEV", "LATE_NEW", "pTDP43_DG_NCI_YN")

# Titles for the plot variables
plot_vars_titles <- c("Age at death\n(y.o., n=99)", "Sex\n(male, n=99)",
    " Syn GBS\n(n=99)", "Neuronal density CA1\n(per mm2, n=99)",
```

```
    "A  Phases MTL\n(n=98)", "CERAD\n(n=99)", "Braak NFT Stages\n(n=99)",
    "pTau CA1\n(n=95)", "CAA type 1\n(n=99)", "CAA Severity\n(n=99)",
    "LATE-NC\n(n=98)", "pTDP DG\n(n=97)")

# Y-axis limits for the plots
lim <- c(109, 1.19, 25, 300, 4.4, 3.4, 6.4, 1.19, 3.4, 3.4)

# Determine if the variables are binary
binary_check <- sapply(plot_vars, is_binary, data = cases)

# Split variables into binary and continuous based on the
# check
binary_vars <- plot_vars[binary_check]
cont_vars <- plot_vars[!binary_check]

# Split titles for binary and continuous variables
binary_vars_titles <- plot_vars_titles[binary_check]
cont_vars_titles <- plot_vars_titles[!binary_check]

# Generate summary statistics for the chosen variables for
# three AD groups - Supplementary Table 8
ad_table_summary <- group_summary(cases_AD, group_var, cont_vars,
    binary_vars, round_no = 2)
ad_table_summary <- ad_table_summary[, c("asyn_pattern", plot_vars,
    "n")]
colnames(ad_table_summary) <- c("asyn_pattern", plot_vars_titles,
    "n")
ad_table_summary <- ad_table_summary[, c("asyn_pattern", cont_vars_titles,
    binary_vars_titles, "n")]

# Save the summary statistics to an Excel file
openxlsx::write.xlsx(ad_table_summary, file = paste0(save_path,
    "ST9_summary_AD_", group_var, ".xlsx"), showNA = FALSE)

# Settings for plotting
base_size <- 80
line_width <- base_size/30
summary_size <- base_size/3
boxplot_width <- 0.5
bar_width <- 0.5
line_color <- "grey20"
outlier_size <- base_size/14

# Colors for the plots
lb_colors <- c("steelblue4", "darkorange3", "orange")

# List to store the plots
p <- list()

# Loop through the plot variables to create plots
for (i in seq_along(plot_vars)) {

    # Remove missing data for plotting
```

```
    cases_plot <- na.omit(cases_AD[, c(group_var, plot_vars[i])])

    if (length(unique(cases_plot[[plot_vars[i]]])) > 2) {
        # Create BOXPLOTS for continuous/ordinal variables
        p[[i]] <- ggplot(cases_plot, aes(x = as.factor(.data[[group_var]]),
            y = as.numeric(.data[[plot_vars[i]]]), fill = as.factor(.data[[group_var]]))) +
            geom_boxplot(size = line_width, width = boxplot_width,
                color = line_color, outlier.size = outlier_size) +
            theme_minimal(base_size = base_size) + theme(axis.title.x = element_blank(),
            axis.title.y = element_blank(), axis.ticks.x = element_blank(),
            axis.text.x = element_blank(), legend.position = "none",
            plot.title = element_text(hjust = 0.5, vjust = -0.2)) +
            ylim(0, lim[i]) + scale_fill_manual(name = "Asyn",
            values = lb_colors) + ggtitle(plot_vars_titles[i])
    } else {
        # Create BARPLOTS for binary variables
        p[[i]] <- cases_plot %>%
            group_by_at(c(group_var)) %>%
            summarise_at(.vars = plot_vars[i], .funs = mean) %>%
            ggplot(aes(x = .data[[group_var]], y = .data[[plot_vars[i]]],
                fill = .data[[group_var]])) + geom_bar(stat = "identity",
            position = "dodge", width = bar_width) + theme_minimal(base_size = base_size) +
            theme(legend.position = "none", axis.title.y = element_blank(),
                axis.title.x = element_blank(), axis.ticks.x = element_blank(),
                axis.text.x = element_blank(), plot.title = element_text(hjust = 0.5,
                  vjust = -0.2)) + scale_fill_manual(name = "Asyn",
            values = lb_colors) + scale_y_continuous(labels = scales::percent_format(accuracy = 1),
            limits = c(0, 1)) + ggtitle(plot_vars_titles[i])
    }
}

# Add margins to the plots
margin = theme(plot.margin = unit(c(t = 4, r = 5, b = 0, l = 2),
    "cm"))

# Save Figure 4
tiff(paste0(save_path, "Figure3_raw.tiff"), units = "in", width = 70,
    height = 34, res = 300, compression = "lzw")
do.call(grid.arrange, c(lapply(p, "+", margin), ncol = 4))
dev.off()

# Results of ANOVA/KW test on three AD groups -
# Supplementary Tables 9,10
results_cont <- perform_anova_KW(cases_AD, cont_vars, group_var)

# Map variable names to titles
results_cont$results_main$variable <- plyr::mapvalues(results_cont$results_main$variable,
    from = cont_vars, to = cont_vars_titles)
results_cont$results_posthoc$variable <- plyr::mapvalues(results_cont$results_posthoc$variable,
    from = cont_vars, to = cont_vars_titles)
results_cont$results_shapiro$variable <- plyr::mapvalues(results_cont$results_shapiro$variable,
    from = cont_vars, to = cont_vars_titles)
```

```
# Save the results to an Excel file
openxlsx::write.xlsx(results_cont, file = paste0(save_path, "ST10_11_results_anova_KW_AD_",
    group_var, ".xlsx"), showNA = FALSE)

# Results of Fisher's test on three AD groups -
# Supplementary Table 11
results_binary <- perform_fishers_test(cases_AD, binary_vars,
    group_var)

# Map variable names to titles
results_binary$results_main$variable <- plyr::mapvalues(results_binary$results_main$variable,
    from = binary_vars, to = binary_vars_titles)
results_binary$results_contingency_tables$variable <- plyr::mapvalues(results_binary$results_contingency[illegible]
    from = binary_vars, to = binary_vars_titles)
results_binary$results_posthoc$variable <- plyr::mapvalues(results_binary$results_posthoc$variable,
    from = binary_vars, to = binary_vars_titles)

# Save the results to an Excel file
openxlsx::write.xlsx(results_binary, file = paste0(save_path,
    "ST12_results_fisher_AD_", group_var, ".xlsx"), showNA = FALSE)
```

Figure 5a, Supplementary Table 12

```
# Load necessary libraries for structural equation modeling
# and plotting
library(lavaanPlot)
library(lavaan)

# Define the structural equation model (SEM)
SEM_model <- "
pTau_positive_neurons_CA1_ST_KG ~ asyn_pattern_amy
LATE_NEW ~ asyn_pattern_amy
Neuronal_Density_CA1_mm2_pTDP_KG ~ pTau_positive_neurons_CA1_ST_KG + LATE_NEW + asyn_pattern_amy"

# Fit the SEM model to the data Note: The data is
# standardized but it doesn't impact the results
fit <- sem(SEM_model, data = scale(cases_AD[, c("asyn_pattern_amy",
    "pTau_positive_neurons_CA1_ST_KG", "LATE_NEW", "Neuronal_Density_CA1_mm2_pTDP_KG")]))

# Get the summary of the fitted model with standardized
# estimates and fit measures
summary_output <- summary(fit, standardized = TRUE, fit.measures = TRUE)

# Plot the SEM model with standardized coefficients and
# significance stars
lavaanPlot(model = fit, coefs = TRUE, stand = TRUE, sig = 0.05,
    stars = c("regress", "covs"), covs = TRUE)

# Convert the summary output to a character vector for
# writing to Excel
summary_lines <- unlist(strsplit(capture.output(summary_output),
```

```r
    "\n"))

# Save results
library(openxlsx)

wb <- createWorkbook()
addWorksheet(wb, "SEM Summary")

for (i in seq_along(summary_lines)) {
    writeData(wb, "SEM Summary", x = list(summary_lines[i]),
        startRow = i, startCol = 1)
}

saveWorkbook(wb, paste0(save_path, "ST13_SEM_details.xlsx"),
    overwrite = TRUE)
```

To obtain the dataset please contact the corresponding author
